# Isolation and characterization of strictly lytic bacteriophages against carbapenem-resistant *Enterobacter cloacae* complex

**DOI:** 10.1128/spectrum.00835-25

**Published:** 2025-10-01

**Authors:** Han-Yueh Kuo, Carl Jay Ballena Bregente, Tran Thi Dieu Thuy, Jazon Harl Hidrosollo, Donna May Dela Cruz-Papa, Tracey Antaeus Gutierrez, Yun-Tsung Huang, Yu-Jui Chuang, Po-Ren Hsueh, Cheng-Yen Kao

**Affiliations:** 1 Division of infectious disease, National Taiwan University Hospital Hsin-Chu Branch 63423 , Hsin-chu, Taiwan; 2 Department of internal medicine, National Taiwan University Hospital Hsin-Chu Branch 569163, Hsin-chu, Taiwan; 3 College of Medicine, National Taiwan University 33561, Taipei, Taiwan; 4 Institute of Microbiology and Immunology, College of Life Science, National Yang Ming Chiao Tung University 390197 , Taipei, Taiwan; 5 College of Medical Technology, Southwestern University PHINMA 657823, Cebu, Philippines; 6 College of Pharmacy and Medical Technology, University of San Agustin 63149, Iloilo City, Philippines; 7 Department of Biological Sciences, College of Science, University of Santo Tomas 37572, Manila, Philippines; 8 Research Center for Natural and Applied Sciences, University of Santo Tomas 37572, Manila, Philippines; 9 Department of Laboratory Medicine, National Taiwan University Hospital, National Taiwan University College of Medicine 38005, Taipei, Taiwan; 10 Department of Medicine, College of Medicine, National Taiwan University 33561, Taipei, Taiwan; 11 Departments of Laboratory Medicine and Internal Medicine, China Medical University Hospital, China Medical University 38019, Taichung, Taiwan; 12 Health Innovation Center, National Yang Ming Chiao Tung University 390197 , Taipei, Taiwan; 13 Microbiota Research Center, National Yang Ming Chiao Tung University 390197 , Taipei, Taiwan; Shenzhen University School of Medicine, Shenzhen, China

**Keywords:** bacteriophage, carbapenem resistance, *Enterobacter cloacae *complex, multidrug resistance, whole-genome sequencing

## Abstract

The global surge of carbapenem-resistant *Enterobacter cloacae* complex (CR-ECC) poses a significant clinical challenge due to limited treatment options. This study aimed to isolate and characterize lytic bacteriophages (phages) targeting CR-ECC. CR-ECC CYEBC080 was used as the bacterial host for isolating lytic phages, and a comprehensive evaluation was conducted on isolated phages, including phage stability under various pH and temperature conditions, host range analysis, killing curves, and therapeutic efficacy in *Galleria mellonella* larvae and a murine bacteremia model. Twelve lytic phages with distinct random amplified polymorphic DNA patterns were isolated, and transmission electron microscopy confirmed their classification under the *Straboviridae* family within the Caudoviricetes class. All phages remained stable across pH 3–11 for up to 90 minutes, with an optimal temperature range of 25°C–37°C. Among them, CYPEBC012 exhibited the broadest host range, lysing 93.75% of 80 CR-ECC isolates, while CYPEBC006 displayed the narrowest, lysing only 65%. Whole-genome sequencing revealed 12 phages with linear double-stranded DNA genomes ranging from 177,624 to 180,648 bp. Phage treatment administered at a multiplicity of infection of 10, 1 hour post-infection, significantly improved larval survival at day 7, reaching ≥80% in most groups, except CYPEBC001 (50%) and CYPEBC004 (60%) treatment groups. In CYEBC080-infected mice, CYPEBC012 treatment resulted in 100% survival by day 3 and 80% survival through day 7. Additionally, phage-treated mice exhibited significantly reduced bacterial loads and high phage titers in blood and liver. This study demonstrates the therapeutic potential of CYPEBC012 as a promising strategy against CR-ECC infections, offering an alternative to conventional antimicrobial treatments.

**IMPORTANCE:**

This study identified and characterized lytic bacteriophages targeting carbapenem-resistant *Enterobacter cloacae* complex, with CYPEBC012 exhibiting the broadest host range and significantly improving survival in a murine bacteremia model. Its stability and efficacy highlight its potential for clinical application. Our findings demonstrate that phage therapy offers a promising alternative to conventional treatments to combat antibiotic-resistant infections.

## INTRODUCTION

Carbapenems are among the most potent antimicrobials for treating severe bacterial infections, especially those caused by multidrug-resistant (MDR) strains. In recent years, the increasing prevalence of carbapenem-resistant Enterobacterales has emerged as a major clinical challenge. As a member of the *Enterobacteriaceae* family, *Enterobacter* warrants particular attention due to its intrinsic resistance to ampicillin and broad-spectrum cephalosporins, significantly limiting alternative antibiotic treatment options (1). The genus *Enterobacter* comprises 27 validly described species, though not all are recognized as human pathogens (2, 3). Among the *Enterobacter* species, *Enterobacter cloacae* and *E. hormaechei*, which are part of the *E. cloacae* complex (ECC), are the most commonly isolated in clinical infections, particularly among immunocompromised patients and those in intensive care units (4).

In recent years, numerous studies have explored the potential of bacteriophages (phages) as an alternative strategy to combat *E. cloacae* infections. The *Enterobacter* phage vB_EcRAM-01 was isolated and identified as a novel member of the family *Straboviridae* and the genus *Pseudotevenvirus* (5). Due to its host specificity, genetic characteristics, and the absence of lysogenic genes, vB_EcRAM-01 presents a promising candidate for various clinical applications, including phage therapy, biological control, and decontamination (5). A recent study on the *Enterobacter* phage vB_EclM_HK6 demonstrated its potential for preventing *E. cloacae* contamination in stored chicken feed samples (6). Phage vB_EclM_HK6 exhibited the broadest host range, infecting 8 out of 16 *E. cloacae* strains and displaying lytic activity against its host strain, *E. cloacae* EC21, at a minimum multiplicity of infection (MOI) of 10^−6^. Furthermore, pre-treatment of raw chicken, chicken nuggets, and pre-prepared cheese salad with vB_EclM_HK6 reduced bacterial counts by 4.6, 2.96, and 2.81 log-units, respectively (6). In Taiwan, a novel *E. cloacae* phage, ECLFM1, was isolated from sewage water using a carbapenem-resistant *E. cloacae* isolate as the host (7). ECLFM1 exhibited rapid adsorption, a short 15 minute latent period, and a burst size of approximately 75 PFU per infected cell. Genomic analysis classified it within the *Karamvirus* genus. Notably, ECLFM1 treatment improved survival in zebrafish infected with *E. cloacae*, highlighting its therapeutic potential (7).

Significant progress has been made in utilizing phage therapy to combat and control MDR organisms. A key advantage of phages in therapeutic applications and biological control is their ability to selectively target bacterial pathogens. Their widespread presence in nature, adaptability, and inherent resilience have shifted them from mere research subjects to promising tools in addressing the growing challenge of antimicrobial resistance. This study focuses on the characterization of lytic phages targeting carbapenem-resistant ECC (CR-ECC) isolates through phenotypic and genotypic assays and *in vivo* infection models.

## MATERIALS AND METHODS

### Bacterial strains and growth conditions

Given the clinical significance of carbapenem resistance as a major challenge in treating bacterial infections, we collected CR-ECC as the target strains for phage isolation and characterization in this study. A total of 80 CR-ECC isolates were collected from bacteremia patients at National Taiwan University Hospital (NTUH) between 2011 and 2020 (listed in Table S1). These isolates were identified by the clinical laboratory through colony morphology, Gram staining, biochemical tests, the Vitek system (bioMérieux, Marcy l’Etoile, France), and Bruker Biotyper Matrix-assisted laser desorption ionization-time-of-flight mass spectrometry. The antibiotic susceptibility of the CR-ECC isolates was evaluated using the disk diffusion method, with the inhibition zone measurements interpreted according to the guidelines of the Clinical and Laboratory Standards Institute (34th edition, 2024). Carbapenemase genes in these isolates were detected using NG-Test CARBA5 (Table S1). *E. hormaechei* CYEBC080 was selected as the host isolate for phage isolation and propagation, while the remaining 79 CR-ECC isolates were tested to assess the host range of the isolated phage’s lytic activity. CYEBC080 was selected due to its representation of a clinically relevant extensively drug-resistant isolate, demonstrating nonsusceptibility to nearly all tested antibiotics, except for amikacin, colistin, and polymyxins. Details of characteristics of *E. hormaechei* CYEBC023 and CYEBC080, used in the mice bacteremia model, are provided in Table S2 and Table S3.

### Phage isolation and purification

Environmental and sewage samples were collected from various locations across Taipei (Beitou district, Northern Taiwan) and Kaohsiung (Southern Taiwan) for phage isolation (Table S4). Ten milliliter of each environmental water sample was subjected to centrifugation at 4,000 × *g* for 10 minutes at room temperature, followed by filtration through a 0.45 µm syringe filter to eliminate large particles and bacteria. The presence of lytic phages was confirmed by observing bacterial lawn clearance using a double-layer agar assay, in accordance with previously published protocols (6, 8). Samples were incubated overnight at 37°C in tryptic soy broth (TSB) alongside the host strain to facilitate phage amplification. Ten consecutive rounds of plaque purification were conducted to guarantee the clonal purity of each phage isolate. The isolated phages underwent a thorough process of purification and enrichment to ensure the production of high-titer phage stocks for further analysis. High-titer phage cultures were preserved in TSB with 50% (wt/vol) glycerol-phage stock and stored at −80°C.

The clonality of phages was subsequently determined using random amplified polymorphic DNA-polymerase chain reaction (RAPD-PCR) with the 1254 primer (5′-CCGCAGCCAA-3′) (9). Phage lysates with titers ≥10⁹ PFU/mL were centrifuged to remove debris. The pellet was treated with TE buffer, SDS, and proteinase K and incubated at 37°C for 48 hours. After salt addition and centrifugation, the supernatant was collected, and DNA was precipitated using cold ethanol and sodium acetate. Following incubation at −80°C, DNA was pelleted, air-dried, and resuspended in sterile water. DNA concentration and purity were measured using a NanoDrop spectrophotometer. Amplification was performed under the following cycling conditions: initial denaturation at 94°C for 5 minutes; followed by 45 cycles of denaturation at 94°C for 30 seconds, annealing at 38°C for 1 minute, and extension at 72°C for 2 minutes; with a final extension at 72°C for 10 minutes. PCR products were resolved on a 1.5% agarose gel stained with ethidium bromide and visualized under UV transillumination.

### Phage host range determination

A modified spot assay was performed, as described in a previous study, to evaluate the host range of the phage against 80 CR-ECC isolates from NTUH (6, 10). To begin, 100 µL of bacteria in the mid-log phase was added to 3 mL of TSB containing 0.7% agar, which was then poured onto tryptic soy agar plates. After 5 minutes of drying period at room temperature, 8 µL of phage suspension (with a titer of >10^8^ PFU) was applied to the surface of the agar. The plates were incubated at 37°C and examined after 16–18 hours for the presence of cleared zones, indicating bacterial lysis caused by the phage. The evaluation of phage susceptibility was based on the extent of clearing observed in the bacterial lawn, following guidelines from a previous study (10).

### Assessment of phage stability at different pH and temperature

The phage survival and stability study under varying temperature and pH conditions was conducted using a spot assay following the previous publication (11). The activity of phages was assessed across various pH (3, 5, 7, 9, and 11). Additionally, the phages were subjected to a temperature gradient (25°C, 37°C, 50°C, 60°C, and 70°C) to analyze the stability of the phages under different conditions. Exposures were conducted at designated intervals (0, 30, 60, and 90 minutes) and were subsequently spot tested against the bacterial host isolate CYEBC080. The results were recorded based on clearing quality, similar to the evaluation method used for host range determination.

### Phage DNA isolation, sequencing, and genome analysis

Phage genomic DNA was extracted from 6 mL of high-titer phage supernatants (>10^8^ PFU) using a commercial phage DNA isolation kit (Norgen Biotek Corp., Canada), following the manufacturer’s instructions. The purity of the extracted DNA was assessed using spectrophotometric measurements at 260 and 280 nm with a Nanodrop 1000 UV-Vis spectrophotometer (Thermo Fisher Scientific, USA) and an Invitrogen Qubit 4 Fluorometer (Thermo Fisher Scientific, USA). Whole-genome sequencing of 12 phages was performed using the Oxford Nanopore MinION MK1C platform (12, 13). Briefly, 1 µg of phage DNA was used to prepare the sequencing library with a ligation sequencing kit (SQK-NBD114.24, Oxford Nanopore Technologies, UK). A total of 300 ng of phage DNA was loaded into the R10.4.1 flow cell. The quality of the generated reads was assessed using FastQC v0.12.0 (https://www.bioinformatics.babraham.ac.uk/projects/fastqc/). Raw signals were base-called into DNA sequences using the Dorado basecalling program (version 0.2.1), and genome assembly was conducted using the Flye *de novo* assembler (version 2.9).

The complete phage genomes were submitted to the National Center for Biotechnology Information (NCBI) database and annotated using Prokka and Rapid Annotations using Subsystems Technology (RAST) (14–16). The quality of the phage genome sequences was assessed using FastQC (Babraham Bioinformatics), which provided an overview of base quality, GC content, and potential sequencing artifacts. The presence of tRNAs within the genome was predicted using tRNAscan-SE (17). To evaluate the safety of the phages for potential therapeutic applications, open reading frames (ORFs) were analyzed using the Virulence Factor Database (18) and the Antibiotic Resistance Gene Database (19). Additional genome analyses and visualizations were conducted using Proksee (20).

A whole-genome phylogenetic tree was constructed to determine the evolutionary relationships among the 22 phages, including the 12 newly isolated in this study. Genomic sequences were aligned and analyzed using the maximum likelihood method implemented in Molecular Evolutionary Genetics Analysis (MEGA, version 11.0) (21), with 1,000 bootstrap replicates to assess the robustness of the inferred clades. In parallel, a genome-wide proteomic tree was generated using ViPTree (Virus Proteomic Tree Server) (22), which performs normalized tBLASTx-based similarity comparisons and constructs phylogenetic relationships based on genome-wide proteomic content. This dual approach enabled both nucleotide- and proteome-level evolutionary comparisons of the phage isolates.

Pairwise average nucleotide identity (ANI) values among the phage genomes were calculated using FastANI (version 1.33) (23). Genome assemblies in FASTA format were used as input. Each genome was segmented into 3,000 bp non-overlapping fragments, which were then compared using a MashMap-based algorithm to rapidly identify high-scoring homologous regions between genome pairs. An identity threshold of 70%, the detection limit of FastANI, was applied. For each genome pair, FastANI computed the ANI value, the number of matched fragments, and the total number of fragments analyzed. Pairs with ANI below 70% or lacking significant homology were reported as empty values and excluded from downstream clustering.

### *In vitro* assessment of the phage bacteriolytic activity

Phage-killing experiments were conducted utilizing the BioTek Synergy HTX multimode microplate reader (CA, United States), employing a flat-bottom 96-well plate (Sarstedt, Germany). Briefly, 100 µL of a 1:100 dilution of overnight cultures, adjusted to an OD_600_ of 0.15 (1 × 10⁶ CFU/mL), was added to a 96-well plate. Phages were prepared in a working solution, beginning at the minimum MOI of 0.001 and increasing to 1,000, and subsequently added to each well. The killing curve assay was performed over 24 hours at 37°C.

### *In vivo* antibacterial activity assessment of lytic phages using *Galleria mellonella* larvae model

To assess the *in vivo* bacteriolytic activity of phages, the *G. mellonella* larvae model was utilized following the protocols established in our previous study (24). A total of 10 larvae, each weighing between 200 and 240 mg, were infected with 10 µL of CYEBC080 at a concentration of 10^6^ CFU under each experimental condition. After 1 hour of bacterial infection, 10 µL of phages (10^7^ PFU) was injected into the left proleg of the larvae using a Hamilton cemented needle syringe. Each experiment was performed in biological duplicate. To evaluate potential mortality caused by the injection or incubation process, a negative control group received 10 µL of phosphate-buffered saline (PBS). The survival of the larvae was monitored every 24 hours for 7 days post-injection. Larvae were considered dead if they failed to respond to light stimulation with a pipette tip.

### Evaluation of phage effectiveness using a bacteremia mouse model

The *in vivo* bacteremia model was established using mice via intraperitoneal inoculation. All animal experiments were approved by the Institutional Animal Care and Use Committee at National Yang Ming Chiao Tung University (NYCU) (approval number 1120802). Female C57BL/6 mice (6 weeks old, 20–22 g) were obtained from the Laboratory Animal Center at NYCU. The mice were assigned to groups of five per condition as follows: Group 1 received only bacterial infection (CYEBC080 or CYEBC023) without treatment, while Groups 2–4 received post-infection phage treatment at an MOI of 10. Bacteria were cultured in TSB at 37°C for 16 hours, then collected by centrifugation at 4,000 × *g* for 10 minutes and resuspended in PBS. Mice were infected with CYEBC080 (8 × 10^8^ CFU) or CYEBC023 (4 × 10^8^ CFU) in 100 µL of PBS, following a previously published protocol with modifications (25). The actual inoculum was confirmed by plating 10-fold serial dilutions on TSB agar. One hour post-infection, the experimental groups received 200 µL of phage (CYPEBC011 and CYPEBC012), while the positive control group received 113 µL of amikacin (6 mg/kg). The negative control group was administered 200 µL of PBS. Infected mice were monitored daily for body weight and survival over 7 days. Additionally, the antibacterial activity of the phages was assessed by quantifying bacterial loads and phage titers. Infected mice were euthanized 16 hours after treatment, and blood, liver, spleen, kidney, and lungs were aseptically collected for bacterial burden analysis.

### Statistical analyses

Statistical analyses were conducted using data from three independent biological experiments. Standard deviations were calculated and reported. One-way ANOVA was employed to evaluate the significance between phages. The Mantel-Cox test was applied to analyze differences in larval and mice survival assays. Results were considered statistically significant when *P* < 0.05. Graphs and statistical analyses were created using GraphPad Prism software (version 10.4.0, GraphPad Software Inc., USA).

## RESULTS

### Isolation and characterization of *E. cloacae* complex phages

The CR-ECC isolate CYEBC080, which demonstrated resistance to 22 out of 25 tested antibiotics and remained susceptible only to colistin, polymyxin B, and amikacin (Table S3), was selected as the host for lytic phage isolation. CYEBC080 had multiple antibiotic resistance genes, including *bla*_ACT-14_, *bla*_CTX-M-15_, and *fosA* located on the chromosome, and *aac (3)-IIa*, *aph (6)-Id*, *aph(3″)-Ib*, *aadA1*, *aac(6′)-Ib-cr*, *bla*_CTX-M-15_, *bla*_TEM-1B_, *bla*_OXA-1_, *bla*_NDM-1_, *bla*_OXA-10_, *catA1*, *catB3*, *qnrB1*, *ARR-3*, *sul1*, *sul2*, *tet(A*), and *dfrA27* on plasmids (Table S3).

After screening over 15 water samples, 18 phages capable of producing clear lytic plaques were isolated using CYEBC080 as the enrichment and permissive host. Based on RAPD fingerprinting analysis, 12 phages displaying distinct patterns were selected for further characterization, while CYPEBC002, CYPEBC005, CYPEBC009, CYPEBC013, CYPEBC020, and CYPEBC021 were excluded (Table S4). The plaque morphology of all selected phages was small and circular, with lytic zone diameters ranging from approximately 0.2 to 1 mm. The phage titers of the isolated phages ranged from 1 × 10⁸ to 8 × 10⁹ PFU/mL at an MOI of 10. The optimal MOI for all phages was determined to range from 0.07 to 6.67 (Table S4).

The morphological characteristics and dimensions of these 12 phages are summarized in Fig. S1 and Table S4. Transmission electron microscope (TEM) analysis, along with genome sequencing, confirmed that all phages belong to the *Straboviridae* family (Fig. S1). Based on TEM images, the phages were classified into three groups according to their head and tail sizes: Group 1 (CYPEBC007, CYPEBC008, CYPEBC012, and CYPEBC018) comprised the smallest phages, with head lengths of 84–99 nm, head widths of 54–79 nm, tail lengths of 109–117 nm, and tail widths of 15–24 nm. Group 2 (CYPEBC001, CYPEBC003, CYPEBC006, CYPEBC011, CYPEBC014, and CYPEBC015) included the largest phages, with head lengths of 104–115 nm, head widths of 76–88 nm, tail lengths of 97–111 nm, and tail widths of 20–29 nm. Group 3 (CYPEBC004 and CYPEBC010) exhibited intermediate sizes, with head lengths of 102–109 nm, head widths of 77–83 nm, tail lengths of 104–108 nm, and tail widths of 20–22 nm. All phages displayed a contractile tail and a prolate head (Table S4).

### Phylogenetic analysis and host range of phages

**Fig 1:**
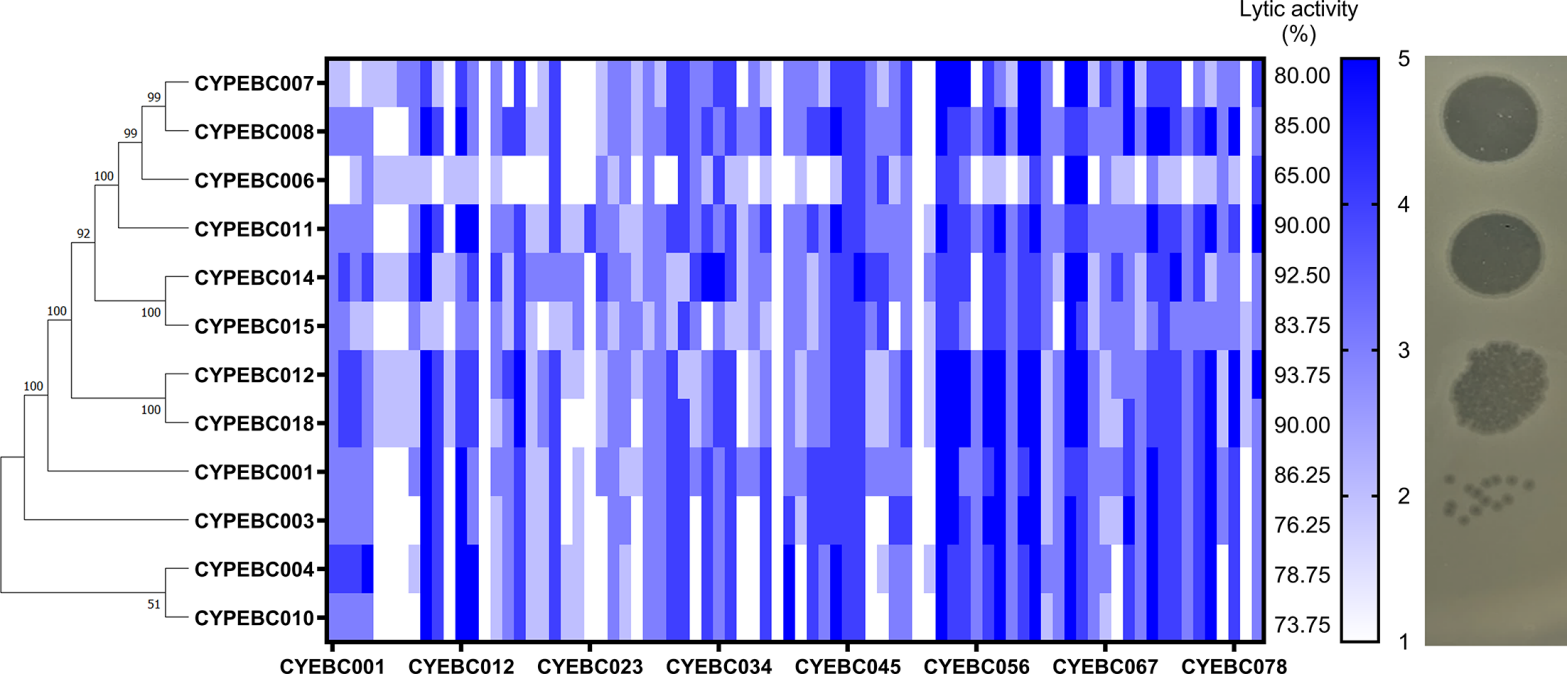
Phylogenetic tree and host range of 12 *Enterobacter* phages. Phylogenetic tree of the 12 *Enterobacter* phages constructed using MEGA (version 11.0) with the Maximum Likelihood method. The numbers on the nodes represent bootstrap support values. The host range of the lytic phages was determined by spot testing against 80 CR-ECC isolates. The assay was performed using the double-layer agar plate method. The intensity of the clearing zone was directly proportional to the phage’s effectiveness in lysing the clinical isolates. Lytic activity was graded as follows: (5) complete clearing, (4) clearing with a faint hazy background, (3) cleared zone with substantial turbidity, (2) few individual plaques, and (1) no clearing.

We further utilized WGS to analyze the genomic characteristics of the 12 phages and constructed a phylogenetic tree. The analysis revealed that the phages clustered into four major groups: CYPEBC007/CYPEBC008, CYPEBC014/CYPEBC015, CYPEBC002/CYPEBC018, and CYPEBC004/CYPEBC010, while CYPEBC001, CYPEBC003, CYPEBC006, and CYPEBC011 exhibited more distant phylogenetic relationships from these clusters (Fig. 1). Notably, all four of these more distantly related phages belonged to morphology group 2 based on TEM observations (Fig. S1 and Table S4).

Nucleotide-based phylogenetic analysis was conducted using MEGA, alongside proteome-based clustering via ViPTree, to clarify the evolutionary relationships among the 12 newly isolated phages (Fig. S2). A notable clustering of CYPEBC phages into monophyletic groups, distinct from established reference phages, suggests the presence of novel phage taxa. Moreover, ANI is a widely accepted and informative metric for assessing phage relatedness, particularly for delineating species boundaries. Therefore, we compared our phage genomes to four well-characterized *Enterobacter* phages, ENC22, Entb_43, fGh-Ecl01, and fGh-Ecl04, using ANI analysis (Table S5). Among these, only *Enterobacter* phage ENC22 showed ANI values above 90% (ranging from 91.5569% to 91.8937%) with our 12 phages, yet still below the 95% species demarcation threshold. These findings indicate that while our phages and ENC22 likely belong to the same genus (*Pseudotevenvirus*, within the family *Straboviridae*), they constitute distinct species. These results suggest that our 12 phages represent a novel species within the *Pseudotevenvirus* genus.

Further screening against 80 clinical CR-ECC isolates demonstrated that among all tested phages, CYPEBC012 exhibited the broadest host range, lysing 93.75% of CR-ECC isolates, whereas CYPEBC006 had the narrowest host range, with only 65% of CR-ECC isolates being susceptible. Within morphology group 2, CYPEBC014 and CYPEBC015 exhibited lysis activity against 92.5% and 83.75% of CR-ECC isolates, respectively. The other group 2 phages, CYPEBC001, CYPEBC003, CYPEBC006, and CYPEBC011, showed lysis activity of 86.25%, 76.25%, 65%, and 90% CR-ECC, respectively (Fig. 1). Within morphology group 1, CYPEBC007, CYPEBC008, CYPEBC012, and CYPEBC018 formed two genetic clusters, each showing lytic activity against 80%–85% and 90%–93.75% of CR-ECC isolates, respectively. In contrast, CYPEBC004 and CYPEBC010, classified as morphology group 3, exhibited high genomic similarity and had relatively lower lytic activity, ranging from 73.85% to 78.75%.

### Stability of phages at different pH and temperature

**Fig 2:**
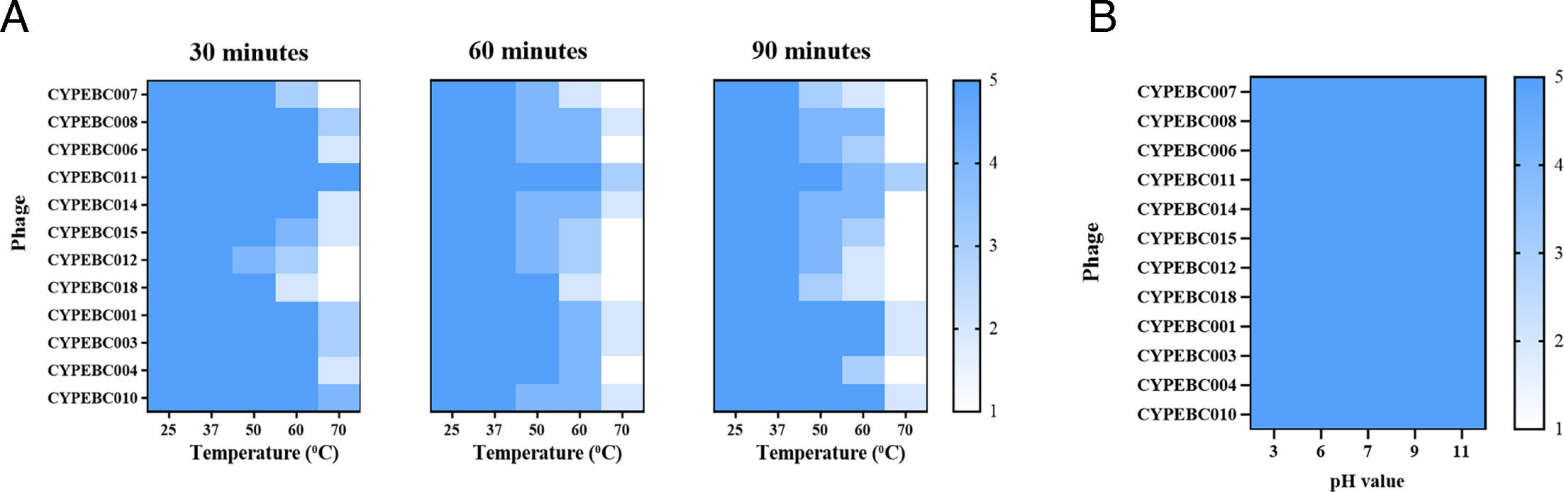
Impact of temperature and pH on phage antibacterial activity. (**A**). Effect of temperature, ranging from 25°C to 70°C for 30, 60, and 90 minutes, on phage activity. (**B**). Effect of pH, ranging from pH 3 to pH 11, on phage activity over 90 minutes. The intensity of the clearing zone was directly related to the phage’s ability to lyse the clinical isolate. Lytic activity was classified as follows: (5) complete clearing (4), clearing with a faint hazy background (3), cleared zone with substantial turbidity (2), few individual plaques, and (1) no clearing.

Results from the temperature stability assays indicated that all 12 phages remained stable at 25°C and 37°C, while their viability progressively declined with increasing temperatures (Fig. 2A). After 90 minutes of incubation at 70°C, eight phages were completely inactivated. However, four phages, CYPEBC001, CYPEBC003, CYPEBC010, and CYPEBC011, retained some activity, though with reduced infectivity. Notably, CYPEBC011 exhibited sustained activity even after 30 minutes at 70°C (Fig. 2A). Additionally, under pH conditions ranging from 3 to 11, all tested phages demonstrated strong stability and remained active for up to 90 minutes (Fig. 2B).

### Antibacterial activity of *Enterobacter* phages

**Fig 3:**
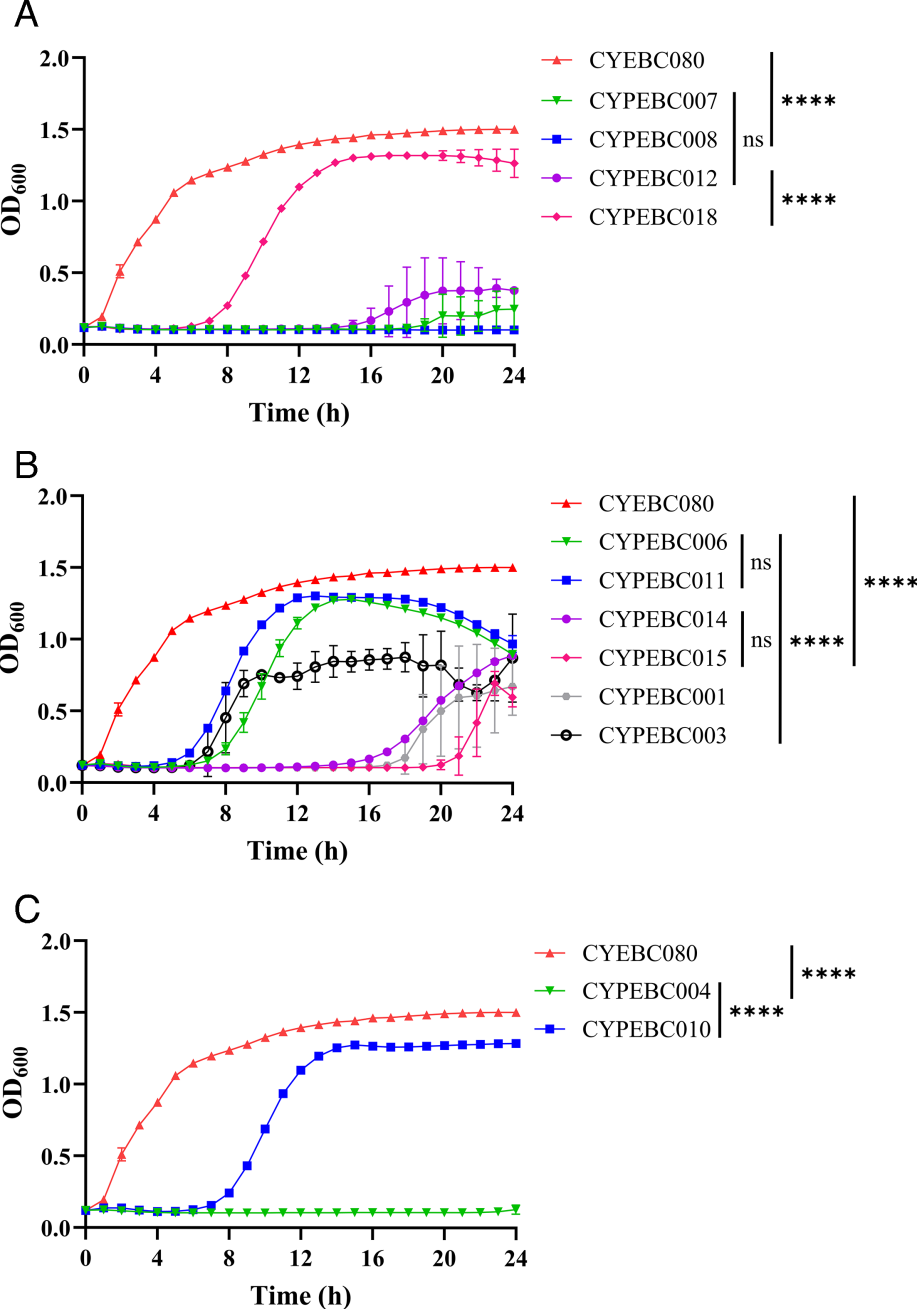
Growth inhibition effects of 12 lytic phages against CYEBC080**.** The red line represents the bacterial growth curve of CYEBC080 incubated at 37°C without phage treatment (as a control group). The others illustrate the reduction in bacterial growth following the treatment of lytic phages, showing significant inhibition of bacterial growth. The data are presented separately based on the different morphological groupings in Table 1. Results are expressed as means ± SD from three independent experiments. A one-way ANOVA test was performed, and differences were considered statistically significant at ****P < 0.0001; ***P < 0.001; **P < 0.01; *P < 0.05; ns, not significant.

We further investigated the antibacterial ability of the 12 phages against CYEBC080. The results showed a progressive decline in OD_600_ in the phage-treated bacterial culture compared to the control culture (without phage treatment) (Fig. 3). Despite using the same MOI of 10, different phages exhibited varying levels of bacterial inhibition. Among morphology group 1 phages, CYPEBC007 and CYPEBC008 nearly eradicated the bacterial host within 24 hours of incubation (Fig. 3A), aligning with genomic phylogenetic data that indicated a high degree of similarity between these two phages. Similarly, CYPEBC018 demonstrated strong inhibitory effects against CYEBC080, whereas CYPEBC012 was only able to suppress bacterial growth for approximately 8 hours (Fig. 3A).

For morphology group 2, phages CYPEBC006 and CYPEBC011 effectively eliminated bacteria for up to 4 hours, after which bacterial growth resumed, leading to a final OD_600_ of 1, similar to the control culture’s OD_600_ of 1.4 (Fig. 3B). However, within the same group, CYPEBC014, CYPEBC015, and CYPEBC001 exhibited stronger inhibition of CYEBC080, though bacterial regrowth was observed after 16 hours (Fig. 3B). Among group 3 phages, CYPEBC004 displayed excellent bactericidal activity against CYEBC080, whereas CYPEBC010 showed noticeable bacterial regrowth as early as 8 hours post-incubation. Although genomic analysis revealed a high degree of similarity between these two phages (Fig. 1), along with comparable host ranges, their ability to inhibit CYEBC080 differed significantly.

We further evaluated the lytic activity of isolated phages across a range of MOI values, from 0.001 to 1,000, to assess the impact of MOI on phage efficacy (Fig. S3). For the majority of phages, no significant differences in bacterial killing were observed across the tested MOIs, indicating consistent lytic performance irrespective of MOI. However, phages CYPEBC014 and CYPEBC011 exhibited MOI-dependent differences in activity, suggesting variable infectivity dynamics or replication efficiency under differing phage-to-host ratios (Fig. S3).

To investigate whether the bacterial regrowth observed during *in vitro* phage treatment was associated with the emergence of phage-resistant mutants, we conducted a phage resistance assessment. Using CYEBC080 as the host strain, we recovered 20 bacterial colonies following exposure to each of the 12 phages and subsequently re-exposed them to their respective phages. Notably, all colonies previously treated with the two phages employed in the *in vivo* animal model, CYPEBC011 and CYPEBC012, exhibited relative OD values below 50%. This result indicates full susceptibility and no detectable resistance, suggesting that the observed regrowth was likely transient and not due to the development of stable phage resistance (Fig. S4).

### Genomic characteristics of 12 *Enterobacter* phages

**Fig 4:**
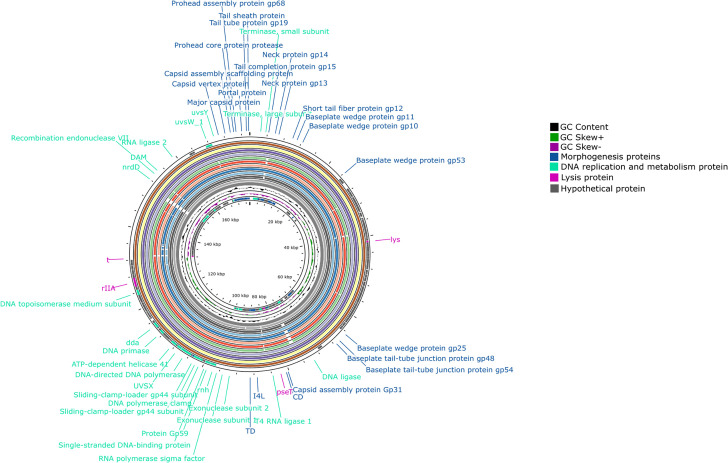
Circular map of the 12 *Enterobacter* phage genomes and the EC-F2 genome. The innermost ring represents the backbone of the CYPEBC007 phage genome, followed sequentially by the genomes of CYPEBC008, CYPEBC006, CYPEBC011, CYPEBC014, CYPEBC015, CYPEBC012, CYPEBC018, CYPEBC001, CYPEBC003, CYPEBC004, and CYPEBC010. The adjacent red ring represents the genome of *Enterobacter* phage EC-F2, which shares over 96% sequence similarity with the genomes of the 12 phages isolated in this study. The outermost ring illustrates the annotated coding regions, with colors denoting their predicted functions as indicated by the color key (in the right corner). Labels for hypothetical proteins (gray arrows) were omitted due to space limitations. The GC content is shown in the black ring, and the inner rings represent GC skew (+, green; −, purple).

The whole-genome sequences of the 12 phages revealed that their genomes are composed of linear double-stranded DNA, with lengths ranging from 177,624 to 180,648 bp and GC content between 44.7% and 44.8% (Table 1). These genomes contain between 271 (CYPEBC018) and 301 (CYPEBC012) ORFs. The functional proteins were classified into three categories, including morphogenesis, DNA replication and metabolism, and host lysis (Fig. 4**;** Table S6). A BLAST comparison with the *Enterobacter* phage EC-F2 (nucleotide accession number MN508624) in the NCBI genome database revealed high sequence similarity with these 12 genomes, ranging from 96.77% (CYPEBC008) to 97.62% (CYPEBC004). Comparable similarities were also observed with phages infecting *Cronobacter* spp., *Citrobacter* spp., and *Klebsiella* spp. Interestingly, all these similar phages belong to the *Straboviridae* family in the class *Caudoviricetes* (tailed phages) (data not shown). No virulence genes or antibiotic resistance genes were identified in the genomes of the 12 phages examined. RAST and Prokka genomic annotation revealed the absence of lysogeny-associated genes, including integrase, repressor, and excisionase, across all 12 phage genomes, supporting their classification as strictly lytic phages.

**TABLE 1:** Genomic characteristics of 12 *Enterobacter* phages

Phage ID	Size (bp)	GC content (%)	ORF (n)^*b*^	tRNA genes (n)	Nanopore sequencing results				Similarity^*a*^ (%)
					Coverage	Total reads (n)	Total bases (mbp)	N50 (bp)	
CYPEBC007	177,922	44.8	280	0	25	46,039	26.7	56,545	97.59
CYPEBC008	178,881	44.7	277	0	416	84,241	86.2	80,468	96.77
CYPEBC006	178,415	44.7	282	0	34	41,034	45.6	98,729	97.40
CYPEBC011	180,648	44.7	278	2	441	17,852	85.4	74,788	97.16
CYPEBC014	178,309	44.7	273	2	1,099	70,703	217.1	132,825	97.61
CYPEBC015	177,624	44.8	273	2	888	76,100	181.7	129,473	97.35
CYPEBC012	179,110	44.7	301	2	1,056	134,471	230.7	95,167	96.85
CYPEBC018	177,637	44.7	271	2	874	125,961	187.1	111,908	97.51
CYPEBC001	178,587	44.7	280	2	1216	63,327	237.8	134,135	97.50
CYPEBC003	177,919	44.8	278	2	535	111,054	108.1	85,912	97.59
CYPEBC004	177,924	44.8	279	2	159	33,423	32.3	48,759	97.62
CYPEBC010	177,921	44.8	282	2	918	48,383	178.8	148,772	97.59

^*a*^ Genome sequence similarity was assessed by performing a BLASTn comparison with Enterobacter phage EC-F2 (NCBI nucleotide accession number MN508624) in the NCBI genome database.

^*b*^ No virulence genes or antibiotic resistance genes were detected in the genomes of these 12 phages using the Virulence Factor Database and ResFinder.

### Therapeutic efficacy of phages against CR-ECC in *G. mellonella* larvae

We utilized the larvae model to evaluate the therapeutic potential of all 12 phages, categorized into three groups based on morphology, against CYEBC080 infection. As shown in Fig. S5, the bacteria infection group without treatment exhibited rapid mortality, with 50% of larvae dying by day 1 and complete mortality by day 2. Phage treatment administered 1 hour post-infection at an MOI of 10 significantly improved larval survival, with survival rates reaching ≥80% after seven days for phages in groups 1, 2, and 3, except for CYPEBC001 in group 2 (50% survival rate) and CYPEBC004 in group 3 (60% survival rate) (Fig. S5).

### Therapeutic efficacy of phage against CR-ECC in a bacteremia mouse model

**Fig 5:**
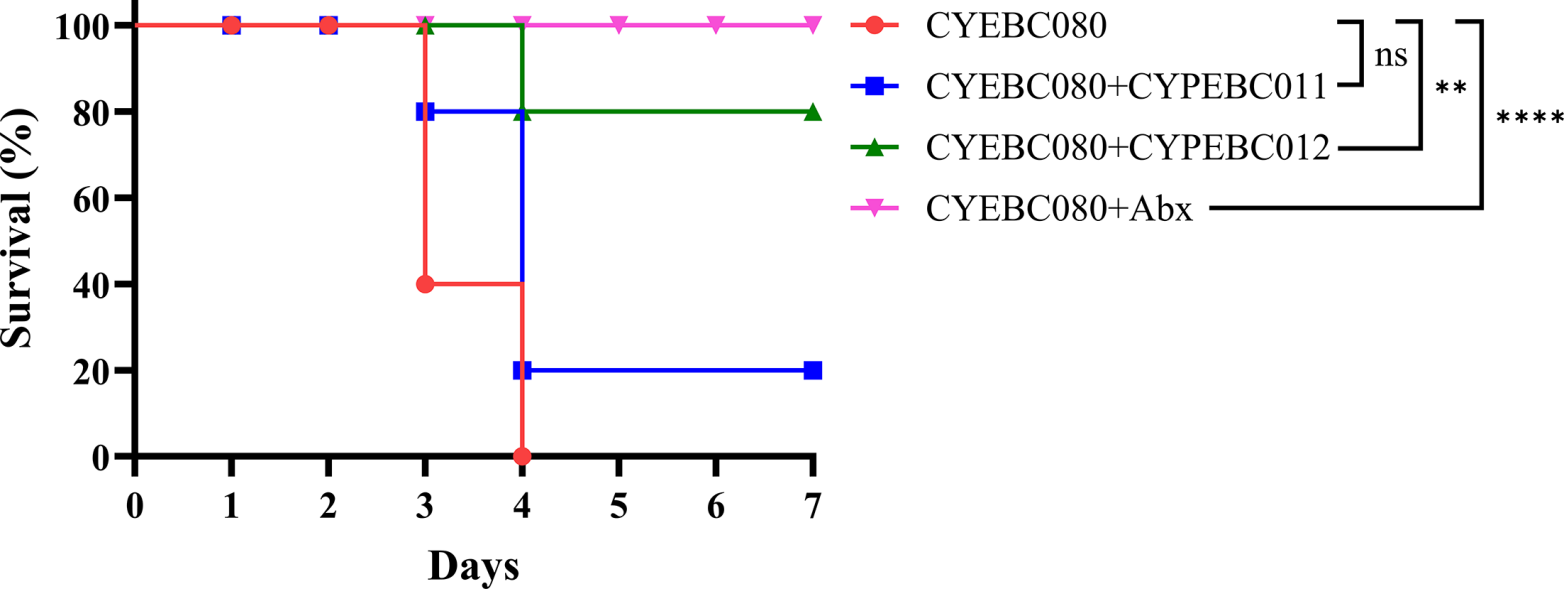
Therapeutic effects of *Enterobacter* phages CYPEBC011 and CYPEBC012 in a bacteremia mouse model infected with CYEBC080. Mouse survival was monitored for up to seven days (5 mice/group). The CYEBC080 group served as the control (no treatment). Phage treatment groups received a dose at MOI 10, administered 1 hour post-infection with CYEBC080. The CYEBC080 +Abx group received amikacin treatment (6 mg/kg) 1 hour after infection. Statistical significance was assessed using the Log-rank (Mantel-Cox) test (****P < 0.0001; **P < 0.01; ns, not significant).

**Fig 6:**
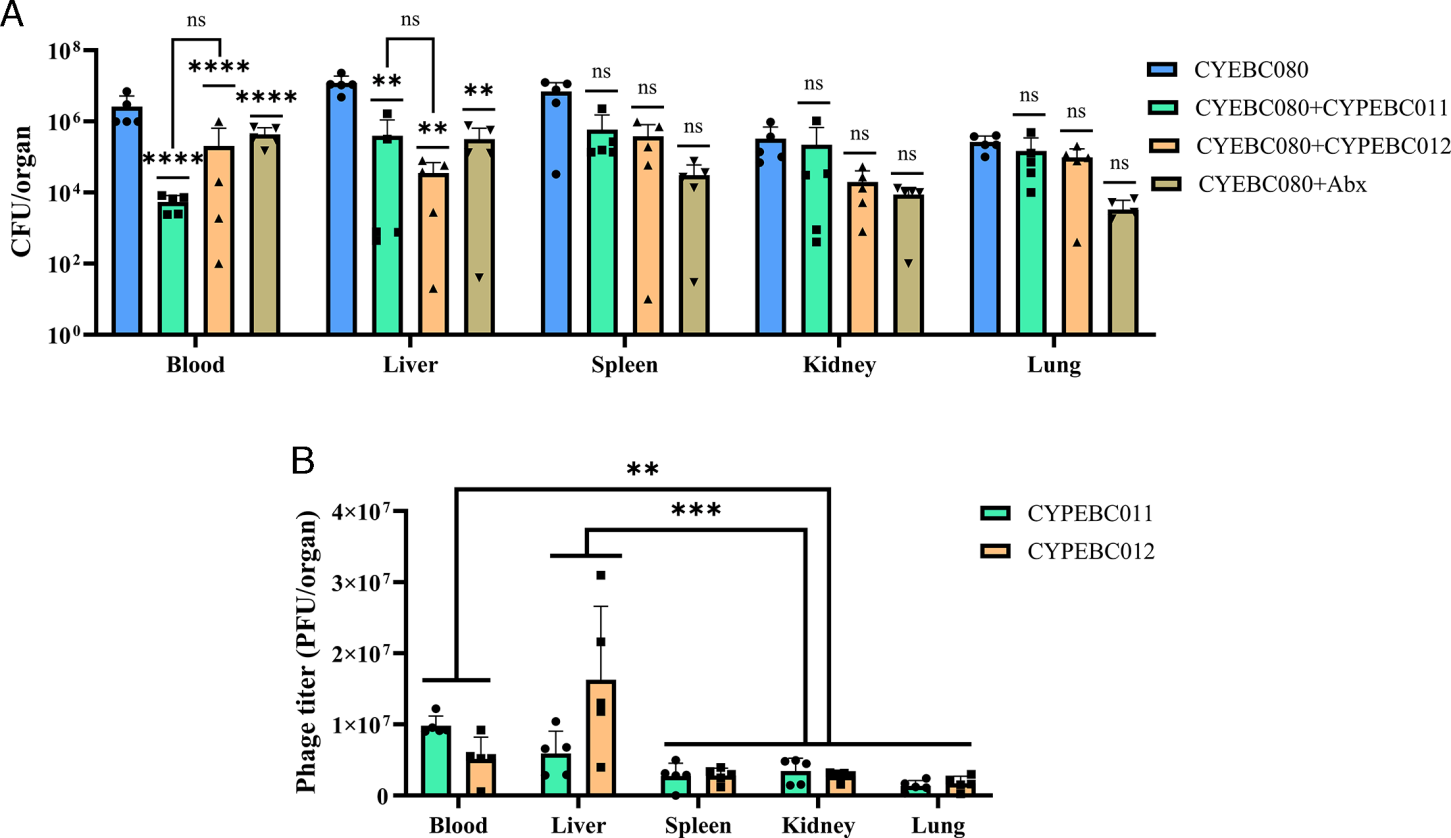
Bacterial burden (**A**) and phage titers (**B**) in blood and organs of CYEBC080-infected mice following 16 hours of treatment with CYPEBC011 and CYPEBC012. C57BL/6 mice (*n* = 5 per group) were intraperitoneally inoculated with CYEBC080. The untreated CYEBC080 group served as the control. For the phage treatment groups, mice received CYPEBC011 or CYPEBC012 at an MOI of 10, 1 hour post-infection. The CYEBC080 +Abx group received amikacin treatment (6 mg/kg) at the same time point. Mice were sacrificed 16 hours after treatment, and bacterial burden and phage titers were analyzed. Statistical comparisons of phage concentrations were performed using one-way ANOVA with Tukey’s multiple comparisons test. Significant differences are indicated by asterisks (****P < 0.0001; ***P < 0.001; **P < 0.01; ns, not significant).

We further assessed the efficacy of phage treatment using a bacteremia mouse model. Mice infected with 4 × 10⁸ CFU of CYEBC080 survived throughout the 7-day observation period. In contrast, those infected with 8 × 10⁸ CFU of CYEBC080 all died by day 4 (Fig. S6A). Based on the host range assay, we identified two phage candidates for the animal model, CYPEBC012, which exhibited the broadest activity by targeting 93.75% of the 80 CR-ECC isolates, and CYPEBC011, the only phage capable of targeting the hypervirulent CYEBC023 (Fig. 1). We then evaluated the therapeutic efficacy of CYPEBC011 and CYPEBC012 as antibacterial agents by assessing survival rates (Fig. 5) and bacterial loads (Fig. 6) in a bacteremia mouse model infected with CYEBC080. The results indicated that mice in the bacterial infection group (without treatment) had 60% mortality by day 3 and 100% by day 4 post-infection (Fig. 5). In contrast, mice infected with CYEBC080 and treated with phage CYPEBC012 at MOI 10, 1 hour post-infection, exhibited a 100% survival rate on day 3 and maintained an 80% survival rate through day 7. This treatment efficacy was comparable to the amikacin treatment group, where all mice survived until day 7. Meanwhile, mice treated with phage CYPEBC011 showed 100% survival on day 2; however, by day 7, only 20% of the mice remained alive (Fig. 5).

We assessed bacterial burden and phage titers in the blood, liver, spleen, kidneys, and lungs of mice from each group 16 hours post-infection. The bacterial load in the blood and liver of the phage-treated groups showed a significant reduction (*P* < 0.05) compared to the untreated bacterial infection group (Fig. 6A). Notably, the lowest bacterial load in the blood was observed in mice treated with CYPEBC011 (5.4 × 10³ CFU), while in the lungs, mice treated with CYPEBC012 exhibited the lowest bacterial burden (3.5 × 10⁴ CFU). However, no significant reduction in bacterial burden was observed in other organs, including spleen, kidney, and lung, which was consistent with the phage titers detected across different organs (Fig. 6B). High phage titers were specifically detected in the blood and liver, with CYPEBC011 reaching 9.8 × 10⁶ PFU and CYPEBC012 reaching 1.3 × 10⁷ PFU.

We further evaluated the effect of phage treatment on another CR-ECC isolate, CYEBC023, in the bacteremia murine model. This isolate exhibited stronger biofilm formation than CYEBC080 (data not shown) and demonstrated resistance to 20 out of 25 tested antibiotics (Table S3). Whole-genome analysis identified several virulence genes in CYEBC023, including *iroN*, *nlpI*, and *terC* (Table S2), which contribute to its ability to infect hosts (*nlpI*) and evade immune defenses through mechanisms such as iron sequestration (*iroN*) and resistance to tellurium ion stress (*terC*). Compared to CYEBC080, CYEBC023 exhibited higher virulence in mice (Fig. S6), despite the fact that CYEBC080 harbors the heat-stable toxin gene *astA*, which is absent in CYEBC023. All mice infected with 4 × 10⁸ CFU of CYEBC023 succumbed by day 2 (Fig. S6B). In contrast, all mice infected with the same CFU of CYEBC080 survived after 7 days, confirming that CYEBC023 exhibits higher virulence than CYEBC080 (Fig. S6A). However, mice infected with CYEBC023 or CYEBC080 had significant body weight loss 7 days post-infection (data not shown).

We evaluated the therapeutic efficacy of phage CYPEBC011 in CYEBC023-infected mice (Fig. S7). Compared to the CYEBC023-only group, which exhibited 60% mortality by day 1 and 100% by day 3, the CYEBC023 + CYPEBC011 group showed 100% survival on day 1, but only 20% survival by day 3 (Fig. S7A). In contrast, all mice in the CYEBC023 + Abx group (treated with amikacin) survived throughout the experiment. We also assessed bacterial burden in CYEBC023-infected mice. Compared to CYEBC080-infected mice, which had 2.6 × 10⁶ CFU in the blood (Fig. 6A), CYEBC023-infected mice exhibited a significantly higher bacterial load, reaching 2.8 × 10⁸ CFU in the blood (Fig. S7B). Although the reduction in bacterial burden following phage and amikacin treatment was not statistically significant, CFU levels in the blood, liver, spleen, kidney, and lungs were generally lower than those in the untreated group, except for the spleen in the phage-treated group. Similarly, phage titers were highest in the blood (1.3 × 10⁶ PFU) and lowest in the kidney (1.5 × 10⁴ PFU) (Fig. S7C).

## DISCUSSION

ECC are common pathogens in healthcare settings, responsible for infections such as pneumonia, urinary tract infections, and septicemia (26, 27). The increasing prevalence of multidrug resistance, particularly carbapenem resistance, has raised significant clinical concerns. In Taiwan, ECC isolates exhibit higher resistance to ertapenem compared to *Escherichia coli* and *Klebsiella pneumoniae*. Among carbapenem-non-susceptible ECC isolates, the most frequently identified species is *E. hormaechei* subsp. *hoffmannii*, followed by *E. hormaechei* subsp. *steigerwaltii* (26). This highlights the urgent need for alternative therapies, such as phages targeting CR-ECC, for effective clinical management.

Previous studies have identified and evaluated specific lytic phages targeting clinical isolates of *E. cloacae* (7, 28–31). A recent study in Taiwan demonstrated the lytic activity of *Enterobacter* phage ECLFM1 (7), highlighting its potential as a therapeutic option against *E. cloacae* infections resistant to meropenem and colistin. Phage ECLFM1 belongs to *Tevenvirinae*, a subfamily within the *Straboviridae* family of the class Caudoviricetes, which is the same classification as our 12 isolated phages belong to *Straboviridae*. Whole-genome sequencing revealed that ECLFM1 has a genome size of 172,036 bp with 288 ORFs, demonstrating genetic similarities with our isolated phages. These findings align with previous phage studies and reinforce the potential of phage therapy as a promising strategy for combating antibiotic-resistant pathogen infections. Additionally, phages from the *Straboviridae* family appear to exhibit strong lytic activity against ECC clinical isolates, further supporting their therapeutic potential.

A total of 80 CR-ECC isolates were tested with our 12 phages. Among them, CYPEBC011, CYPEBC014, CYPEBC012, and CYPEBC018 exhibited a host range of ≥90% against the 80 CR-ECC isolates, demonstrating broad-spectrum lytic activity. Previous research has shown that long-tailed phages, particularly those in the Caudoviricetes class, display a broad range of activity against gram-negative bacteria (29, 32, 33). This is due to their diverse receptor-binding proteins, genetic adaptability, and distinct characteristics of their lytic cycles (32, 34). However, the specific receptors that contribute to the differences in host range across our phages in the same *Straboviridae* family remain to be further elucidated.

In contrast to other recent phage studies that typically concentrate on closely related isolates within a single taxonomic group or genus, our collection of 12 phages demonstrates enhanced evolutionary and genomic diversity. Phages are classified into at least two distinct evolutionary lineages, which may encompass different families or genera, as demonstrated by phylogenomic analysis. The CYPEBC phages exhibit well-supported monophyletic clades that are distinct from established reference phages, indicating the existence of novel or under-characterized phage taxa. The observed degree of divergence surpasses that found in conventional therapeutic phage panels documented in earlier research (28, 35) in which isolates typically cluster within a limited phylogenetic range. The taxonomic and genomic diversity significantly increases the therapeutic potential of the new isolated phages. Increased diversity may be associated with a wider host range, diminished redundancy, and enhanced potential for synergistic combinations, all of which are critical in formulating effective phage cocktails targeting MDR pathogens. These findings indicate that phylogenetic heterogeneity improves cocktail efficacy and resilience, as demonstrated by recent research, thereby supporting the inclusion of diverse phages in therapeutic design (36).

Environmental stability is critical when evaluating the suitability of phages for clinical applications. Phage activity tends to decrease at higher temperatures due to protein denaturation and structural instability under prolonged heat exposure (37). The temperature effects showed a decline in phage activity starting at 50°C, with complete inactivation occurring at 70°C after a 90 minute incubation. These results are consistent with findings from previous studies (5, 7). We assessed the stability of our phages under varying pH levels and temperatures. Notably, CYPEBC011 maintained the highest antibacterial activity even after 90 minutes at 70°C, reinforcing its potential for future clinical applications.

Genome analysis combined with phenotypic assays showed that all isolated phages produced clear lysis zones in both spot and plaque assays and effectively suppressed bacterial growth for up to 8 hours in liquid infection assays at an MOI of 10. These findings confirm that all our phages are lytic. They efficiently inhibit CR-ECC growth without displaying cytotoxicity or harboring genes that could facilitate the spread of antibiotic resistance. Interestingly, the majority of the isolated phages displayed MOI-independent killing, suggesting robust lytic efficacy even at low phage-to-host ratios. This behavior is comparable with previously documented characteristics of strictly virulent lytic phages, which include quick adsorption kinetics and huge burst sizes that enable effective bacterial clearance independent of initial MOI (38, 39). Furthermore, Abedon (40) and Nang et al. (41) emphasize that these phages often exceed the replication threshold even with minimal phage inputs, rendering them potent candidates for therapeutic applications when *in situ* phage multiplication is essential.

During the phage lytic cycle, the coordinated action of several key proteins ensures efficient bacterial lysis. Initially, antiholin regulates the timing of lysis by inhibiting holin, preventing premature membrane permeabilization. Once the phage has completed replication, holin forms pores in the bacterial membrane, creating passageways for L-alanyl-D-glutamate peptidase, which degrades the peptidoglycan layer by cleaving the L-alanyl-D-glutamate bond, weakening the bacterial cell wall. Meanwhile, protein rIIA may assist in overcoming host defense mechanisms, ensuring successful phage propagation. This sequential process ultimately leads to bacterial cell lysis and the release of new phage progeny (30, 42, 43). Gene annotation results revealed that lysis-related proteins, including holin, L-alanyl-D-glutamate peptidase, and rIIA, were present in the genomes of all 12 phages, except for CYPEBC010, which lacked rIIA. Additionally, CYPEBC001 and CYPEBC003, which exhibited close genetic relatedness, possessed an extra lysis-related gene, Rz1 (44). However, the presence of Rz1 did not correlate with enhanced *in vitro* bacterial inhibition activity.

The phagenomic database showed that CYPEBC012 has high phage virulence, while CYPEBC011 exhibits medium to low virulence. Consistently, both the killing assay and the murine bacteremia model reveal that CYPEBC012 is more effective at inhibiting CYEBC080 than CYPEBC011, suggesting that CYPEBC012 may serve as a more effective phage therapy than CYPEBC011. However, the potential of phage cocktails made up of different phages, or the combination of phage therapy with antibiotics, as well as the comparison between multi-dose and single-dose treatments used in this study, warrants further investigation in future research.

In this study, we demonstrate the lytic effectiveness of two genetically diverse phages, CYPEBC011 and CYPEBC012, and their potential for treating infections in a mouse bacteremia model. These phages significantly reduce bacterial burden in various organs within 16 hours of administration. Notably, phage titers vary across organs post-exposure, suggesting that organ-specific distribution is strongly influenced by the route of administration. While previous studies have highlighted the emergence of phage resistance following treatment, the use of phage cocktails not only enhances bactericidal efficacy but also mitigates the risk of resistance development (31, 45–47). Interestingly, a prior study reported that phage resistance via lipid A modification may also lead to decreased lipopolysaccharide toxicity (48). These findings further support the potential of phage cocktails as a promising strategy for combating MDR pathogen infections in the future. Importantly, our results suggest that the observed regrowth of bacteria *in vitro* was likely transient in nature, potentially attributable to delayed lysis or temporary physiological states rather than the selection or emergence of phage-resistant mutants.

In conclusion, this study is one of the few investigations assessing the efficacy of *Enterobacter* lytic phages against CR-ECC isolates. We identified two promising phage candidates, CYPEBC011 and CYPEBC012, as potential alternative treatments for CR-ECC bacteremia. However, before further clinical application, additional studies are needed to evaluate the therapeutic efficacy of phage-antibiotic combination and phage-cocktail treatments, as well as to investigate the mechanisms and frequency of ECC resistance to these phages.

## Data Availability

All data generated or analyzed in this study are available within this article and its supplemental materials. Bacterial and phage genome sequences have been submitted to the NCBI database, and the corresponding accession numbers are provided in Table S2 and S4.

## SUPPLEMENTAL MATERIAL

Supplemental material spectrum.00835-25-s0001.docx Supplemental methods; Fig. S1 to S7.

Supplemental tables spectrum.00835-25-s0002.docx Tables S1 to S5.

Table S6 spectrum.00835-25-s0003.xlsx Annotated genomic features of the isolated phages.

ASM does not own the copyrights to Supplemental Material that may be linked to, or accessed through, an article. The authors have granted ASM a non-exclusive, world-wide license to publish the Supplemental Material files. Please contact the corresponding author directly for reuse.
