# Supplementary material for: Isolation and characterization of strictly lytic bacteriophages against carbapenem-resistant Enterobacter cloacae complex
Source: Microbiol Spectr. 2025 Oct 1;13(11):e00835-25. doi: 10.1128/spectrum.00835-25 (PMC12584729; doi:10.1128/spectrum.00835-25)
Supplement: Supplemental material — Supplemental methods; Fig. S1 to S7. [file spectrum.00835-25-s0001.docx]

**Supplementary methods**

**Phage structure observation**

Concentrated phage samples were analyzed using transmission electron microscopy (TEM) to investigate their morphology and structure. Ten microliters of high-titer phage (>10^8^ plaque-forming units (PFU)/mL) were placed on a formvar-coated copper grid (300 mesh copper grids) and stained with 2% uranyl acetate. The morphological features were examined using a JEOL JEM-1400 TEM (JEOL, Tokyo, Japan), following protocols outlined in previous studies (1, 2). The resulting micrographs were analyzed to classify the phage isolates based on the latest morphological criteria set by the International Committee on Taxonomy of Viruses (ICTV) (3).

**Phylogenetic analysis using MEGA (Molecular Evolutionary Genetics Analysis) and VIPtree (Virus Proteomic Tree Server)**

We utilized whole-genome proteomic and gene-based phylogenetic methods to ascertain the evolutionary relationships among our isolated bacteriophages and reference phages. A whole-genome proteomic-based phylogeny was performed utilizing VIPtree, which constructs a proteome-based tree derived from genome-wide tBLASTx similarity scores across viral genomes. The tree output presents percentage similarity metrics and clustering with reference phages in the viral proteomic tree, facilitating genus- and species-level classification. Meanwhile, a nucleotide-based phylogenetic tree was constructed utilizing MEGA (version 11) software. Conserved marker genes, such as the terminase large subunit was aligned utilizing ClustalW, and a Maximum Likelihood tree was constructed with 1000 bootstrap replicates.

***In vitro* assessment of the phage bacteriolytic activity**

Phage-killing experiments were conducted utilizing the BioTek Synergy HTX multimode microplate reader (CA, United States), employing a flat bottom 96-well plate (Sarstedt, Germany). Briefly, 100 µL of a 1:100 dilution of overnight cultures, adjusted to an OD_600_ of 0.15 (1×10⁶ CFU/mL), was added to a 96-well plate. Phages were prepared in a working solution, beginning at the minimum MOI of 0.001 and increasing to 1000, and subsequently added to each well. The killing curve assay was performed over 24 hours at 37°C.

**Phage resistance assessment**

To determine whether the host strain developed resistance following phage treatment, we used CYEBC080 as the host strain. Twenty bacterial colonies were isolated using CYEBC080 as the host strain after exposure to each of the 12 phages and were subsequently re-challenged with their corresponding phages. Phage susceptibility was quantified by measuring the OD of cultures after phage treatment and calculating the relative OD using the following formula: Relative OD (%) = (OD₆₀₀ of phage-treated culture / OD₆₀₀ of untreated control) × 100. Based on the relative OD values, isolates were classified into three categories of phage susceptibility: Strongly susceptible: relative OD < 50%; Partially resistant: relative OD between 50% and 80%; Likely resistant: relative OD > 80% (4, 5).

***In vivo* antibacterial activity of lytic phages using the *Galleria mellonella* larvae model**

To assess the *in vivo* antibacterial activity of lytic phages, we employed the larvae infection model following protocols established in our previous study (6). A total of 10 larvae, each weighing between 200 and 240 mg, were infected with 10 µL of CYEBC080 at 10⁶ CFU under each experimental condition. One hour post-infection, 10 µL of *Enterobacter* phage suspension (10⁷ PFU) was injected into the larvae’s left proleg using a Hamilton syringe. Each experiment was performed in biological duplicate. To evaluate potential mortality due to injection or incubation processes, a negative control group received 10 µL of PBS. Larval survival was monitored at 24-hour intervals over seven days post-injection. Larvae were considered dead if they failed to respond to light stimulation from a pipette tip.

**Evaluation of phage effectiveness against *Enterobacter* using bacteremia mice model**

The *in vivo* bacteremia model was established in mice via intraperitoneal inoculation. All animal experiments were approved by the Institutional Animal Care and Use Committee at National Yang Ming Chiao Tung University (IACUC approval number: 1120802). Five six-week-old female C57BL/6 mice (20–22 g) were obtained from the NYCU Laboratory Animal Center. The mice were divided into five groups, with five mice per group: Group 1: Bacteria-only treatment (CYEBC023 or CYEBC080); Groups 2-4: Post-infection phage treatment (MOI = 10). The bacteria were cultured in TSB broth at 37°C for 16 hours. After centrifugation at 4000 × g for 10 minutes, the bacterial pellet was resuspended in phosphate-buffered saline (PBS). Each mouse was infected with 100 μL of CYEBC023 or CYEBC080 in PBS, with the actual inoculum validated by plating 10-fold serial dilutions on TSB agar. One hour post-infection, the experimental and positive control groups received 200 µL of phage CYPEBC011 (4 × 10⁹ PFU) and 113 µL of amikacin (6 mg/kg), respectively, while the negative control group received 200 µL of PBS. Infected mice were monitored daily for seven days, with survival rates and body weight recorded. To further evaluate phage treatment efficacy, bacterial and phage titers were quantified. Infected mice were euthanized 16 hours post-treatment, and blood, liver, spleen, kidneys, and lungs were aseptically collected for bacterial burden analysis.

**Figure S1**


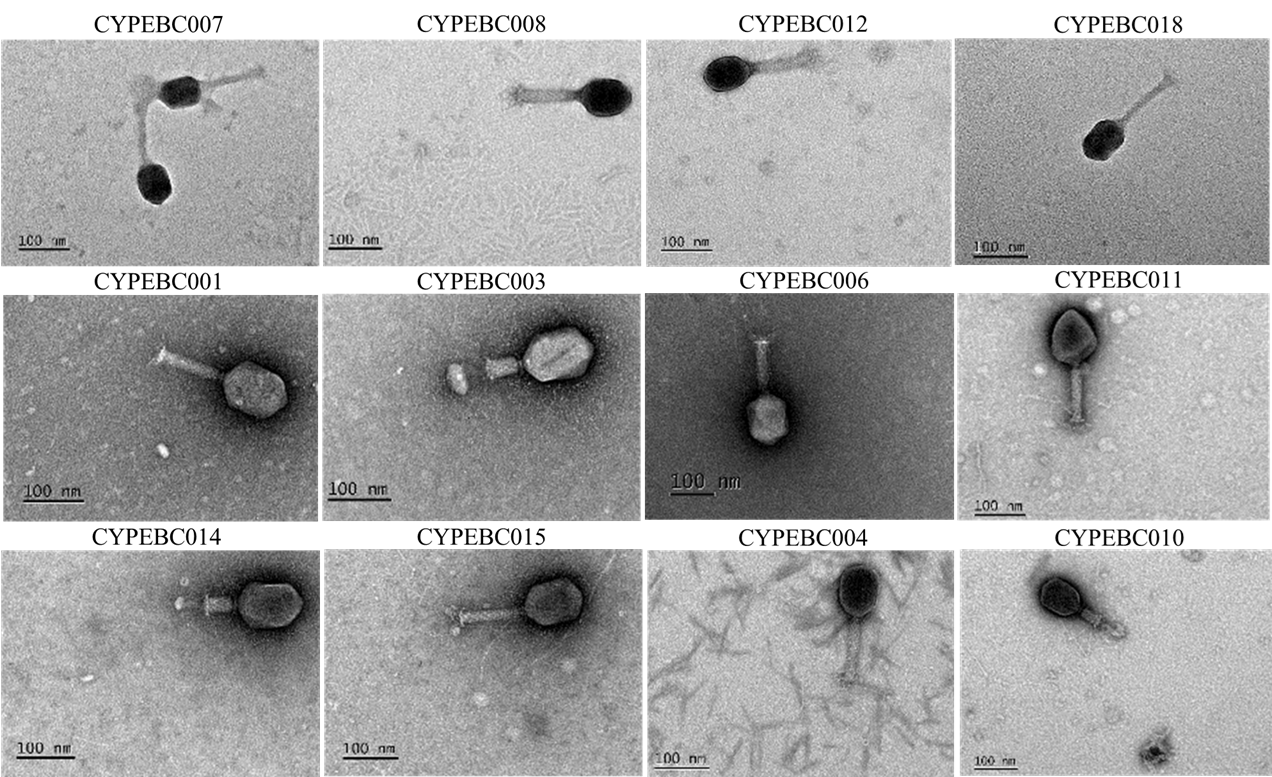


**Fig S1. Transmission electron micrograph images of the isolated phages were captured using a JEOL JEM-1400Plus transmission electron microscope at a magnification of 300,000x.** The scale bar in each image represents 100 nm.

**Figure S2**

**
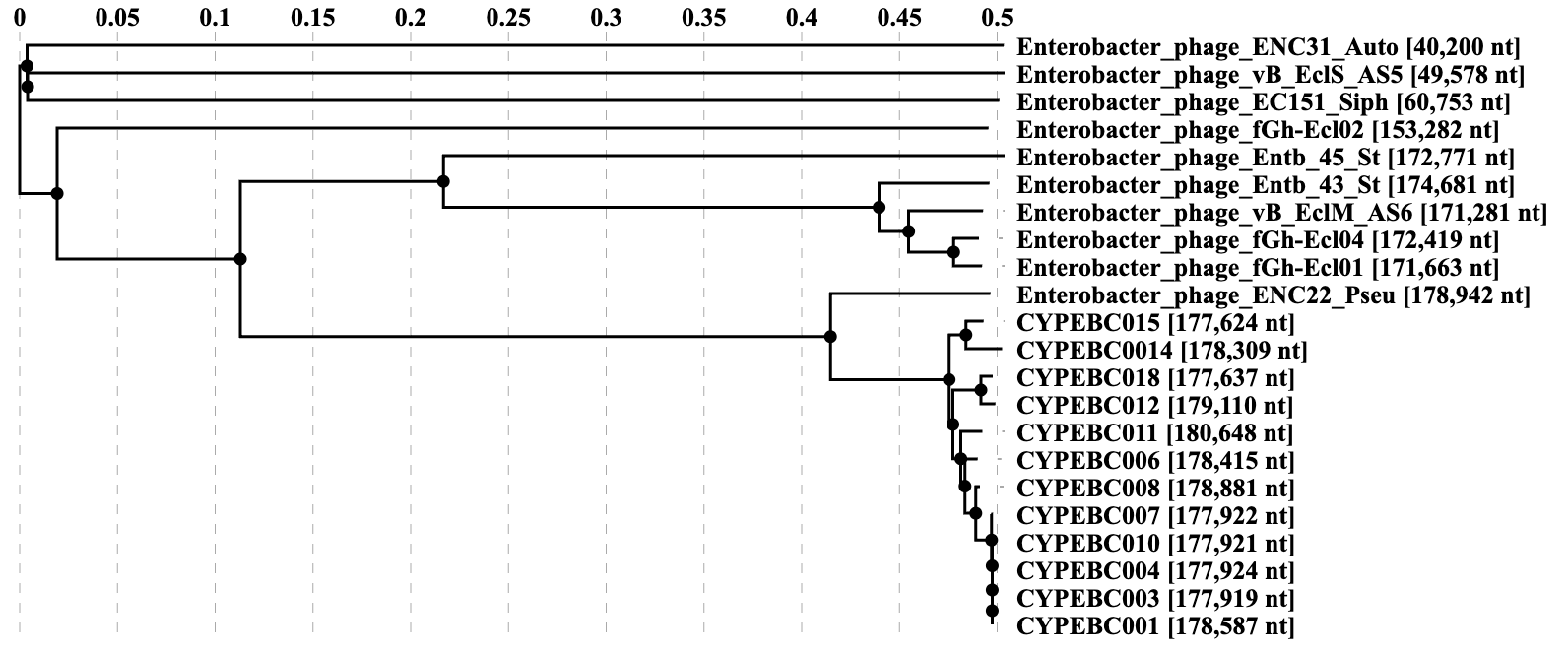
(A).**

**
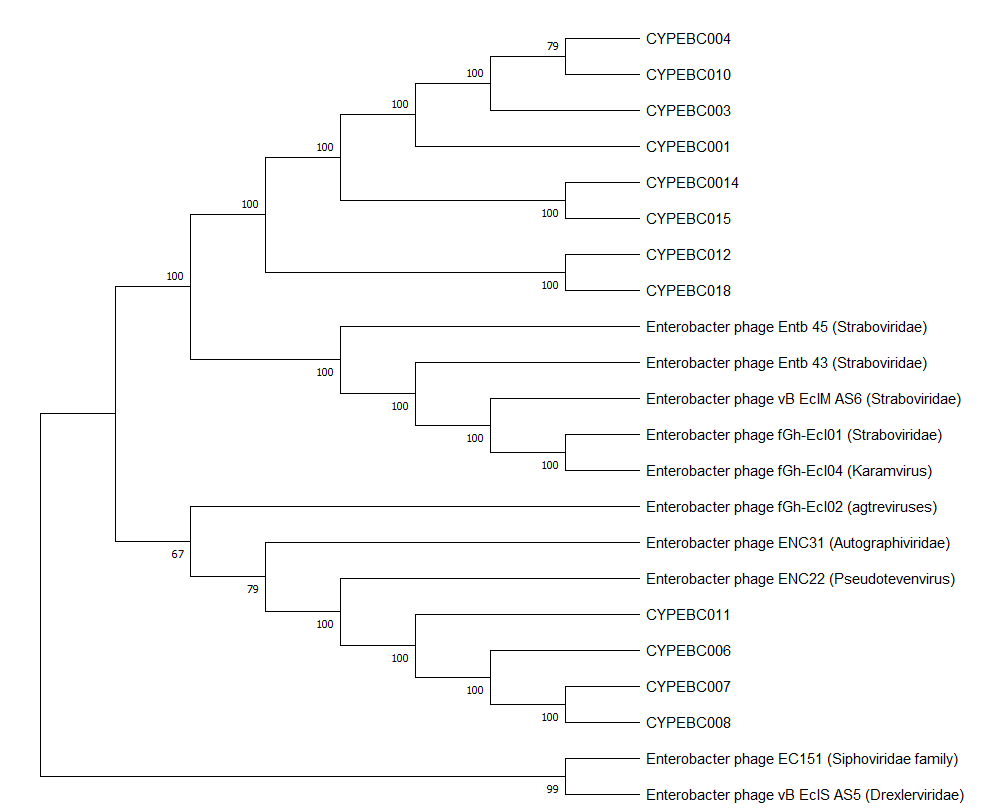
(B).**

**Fig S2. Phylogenetic tree of the 12 phages isolated in this study and 10 phages retrieved from genome databases. (A)** Whole-genome proteomic phylogeny of 22 phages (including our 12 isolated phages) generated by VIPtree (Virus Proteomic Tree Server) based on tBLASTx scores. Phages clustered according to similarity with reference phages in the NCBI viral genome database. **(B)** Maximum likelihood tree constructed in MEGA based on the alignment of the terminase large subunit gene using the Jukes-Cantor model with 1000 bootstrap replicates. The tree reveals fine-scale evolutionary divergence among closely related phages**.** Both phylogenetic trees comprises 22 phages, with 12 newly isolated phages from the current study distinctly labeled, typically using CYPEBC identifiers. The tree illustrates the clustering of CYPEBC phages into two primary clades, indicating phylogenetic diversity among newly isolated phages. Reference phages from established families, including *Straboviridae*, *Siphoviridae*, and *Drexlerviridae*, are incorporated for taxonomic context.


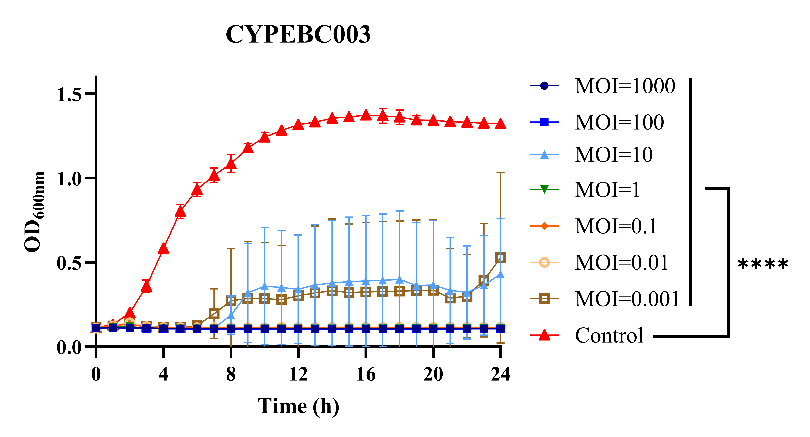

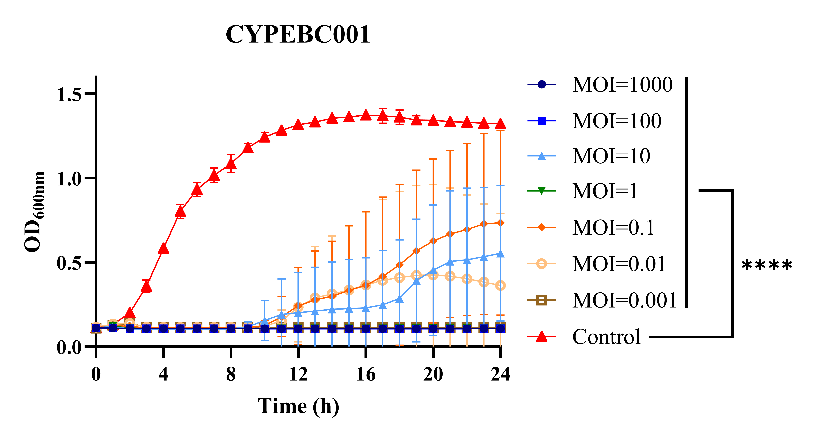
**Figure S3**


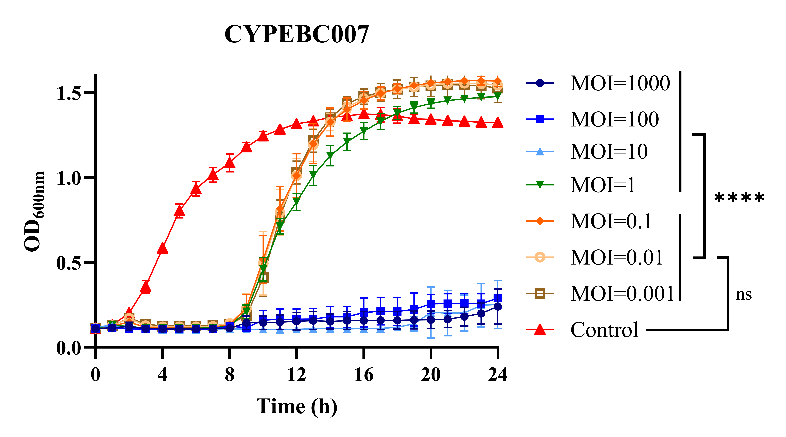

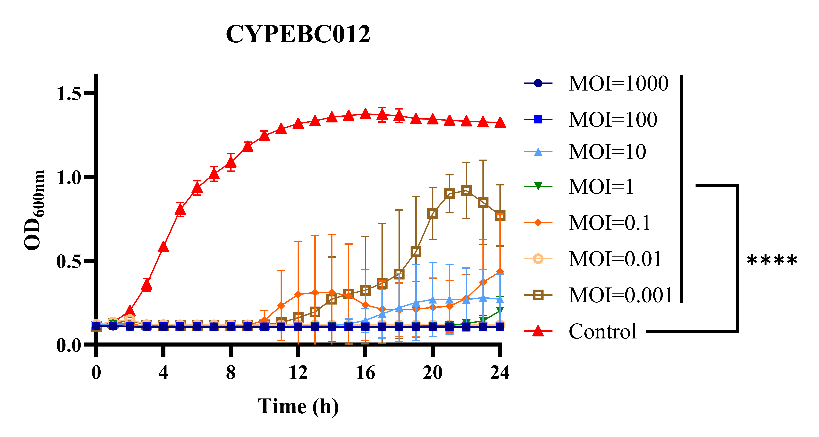

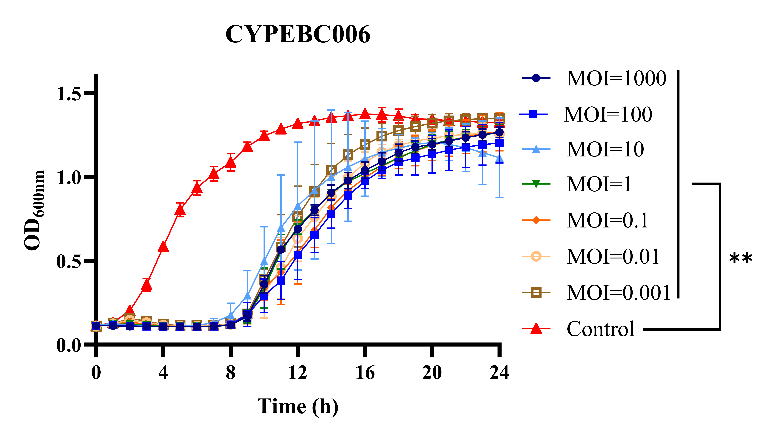

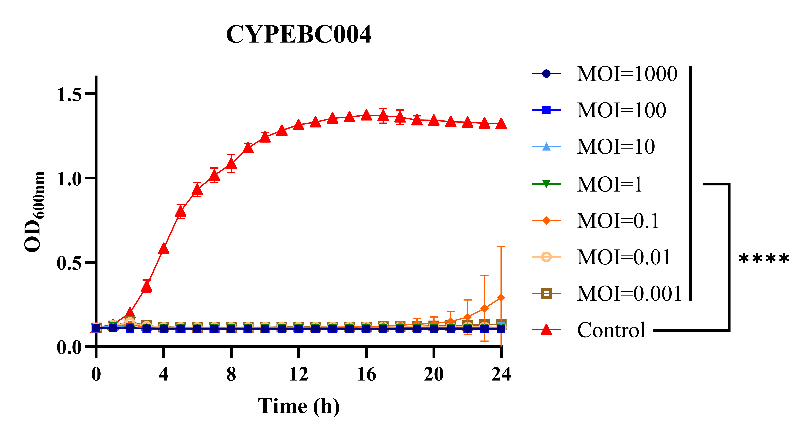


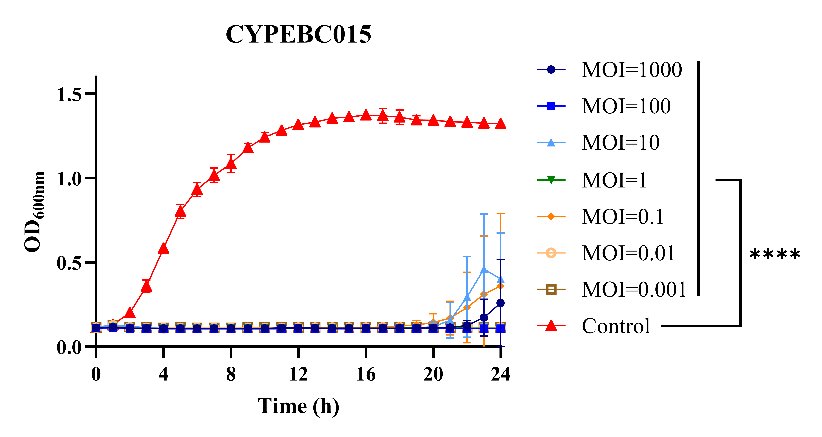

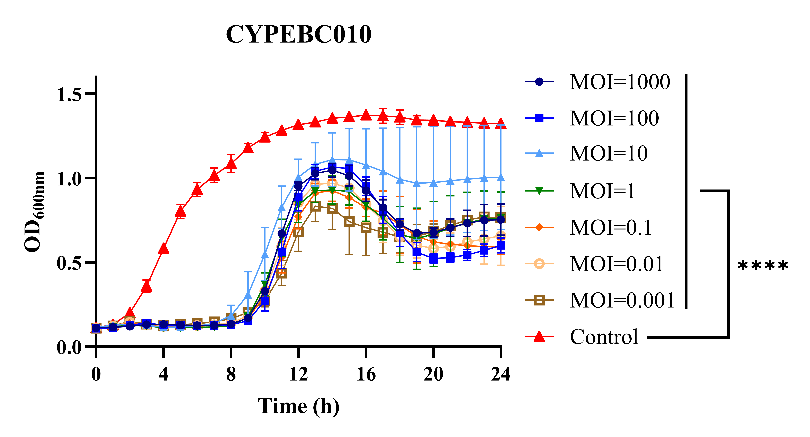


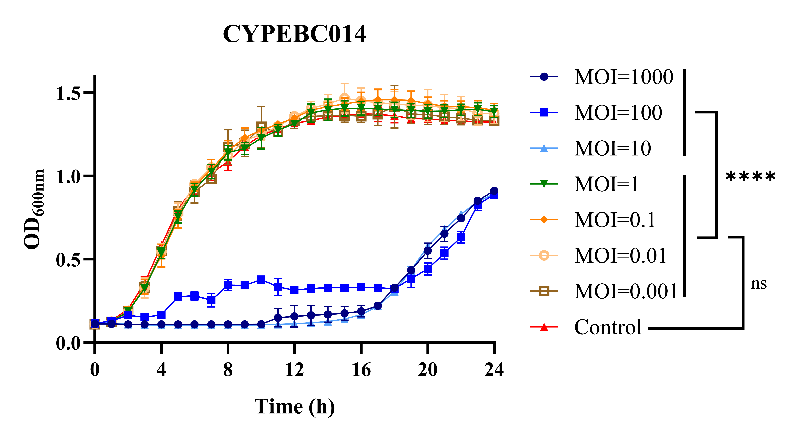

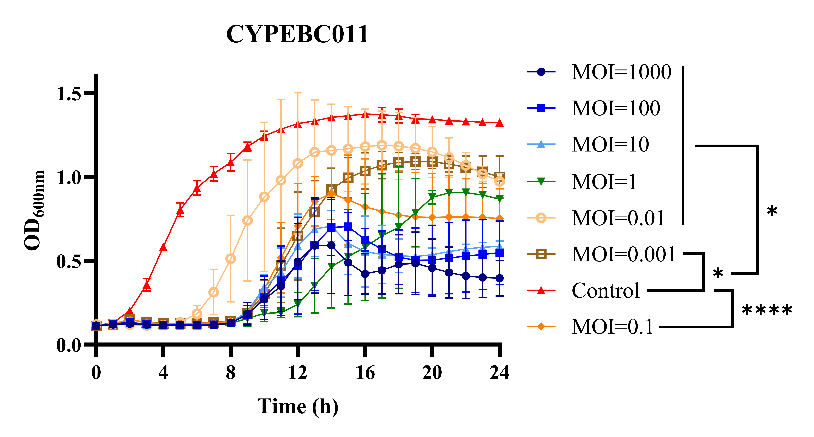

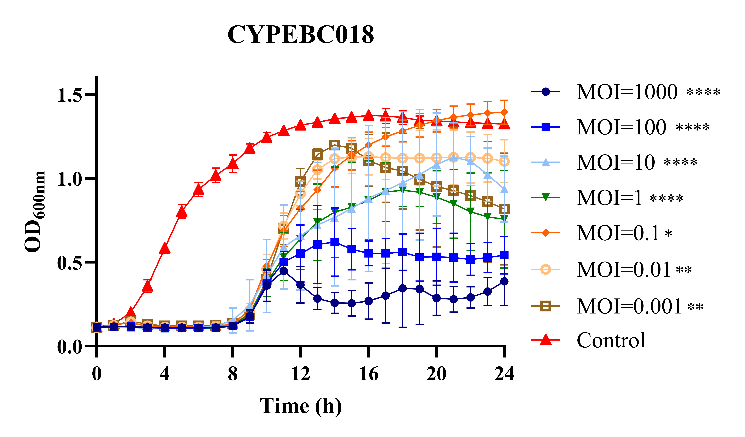

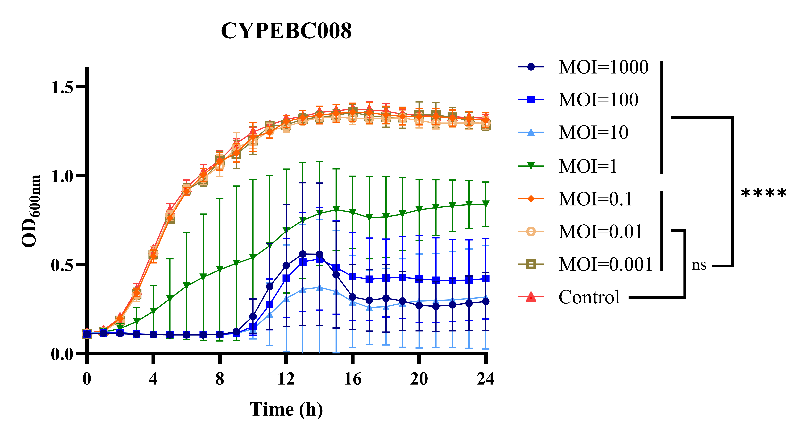


**Fig S3. Growth inhibition effects of 12 lytic phages against CYEBC080 using different MOI.** The red line represents the bacterial growth curve of CYEBC080 incubated at 37°C without phage treatment (as a control group). The others illustrate the reduction in bacterial growth following the treatment of lytic phages, showing significant inhibition of bacterial growth at different MOI. The results for CYPEBC001, CYPEBC003, CYPEBC004, CYPEBC006, CYPEBC012, and CYPEBC015 indicated no variation among the different MOI treatments. In between experiments, CYPEBC007, CYPEBC008, and CYPEBC018 demonstrated a reduction in bacteria from MOI 1000 to MOI 1. CYPEBC014 reduced bacterial counts at a MOI of 1000 to 10. In contrast, CYPEBC011 demonstrated a reduction in bacteria following phage treatment after exposure to a MOI of 1000 to 0.001. Results are expressed as means ± SD from three independent experiments. A one-way ANOVA test was performed, and differences were considered statistically significant at **** *p* < 0.0001; *** *p* < 0.001; ** *p* < 0.01; * *p* < 0.05; ns = not significant.

**Figure S4**

**Fig. S4. Phage resistance assessment of *Enterobacter* CYEBC080 strain.** Twenty bacterial colonies were isolated using CYEBC080 as the host strain after exposure to each of the 12 phages and were subsequently re-challenged with their corresponding phages. Their susceptibility was evaluated based on relative optical density (OD) measurements. Relative OD was calculated as (OD₆₀₀ of phage-treated group / OD₆₀₀ of untreated control) × 100. Strains were classified into three categories: strongly susceptible (<50%, indicating significant lysis), partially resistant (50–80%, suggesting partial or delayed lysis), and likely resistant (>80%, indicating minimal or no lysis).

**Figure S5**

**(A).**


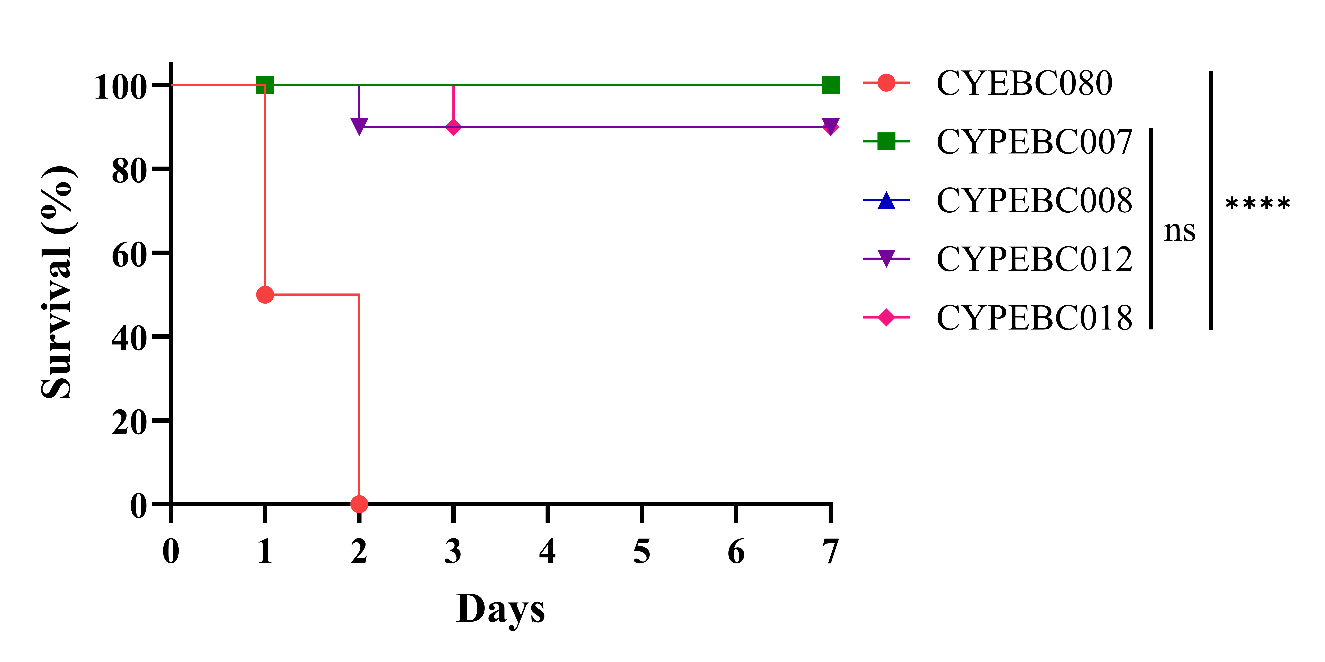


**(B).**


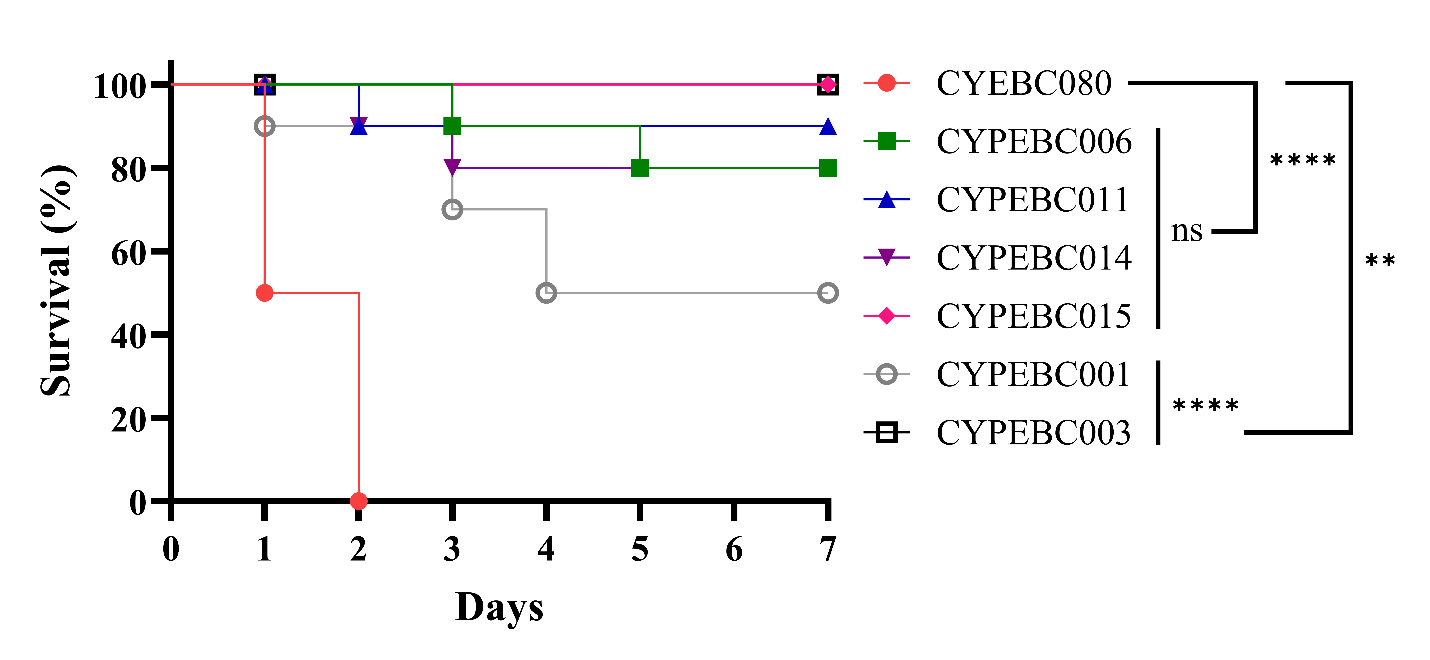


**(C).**


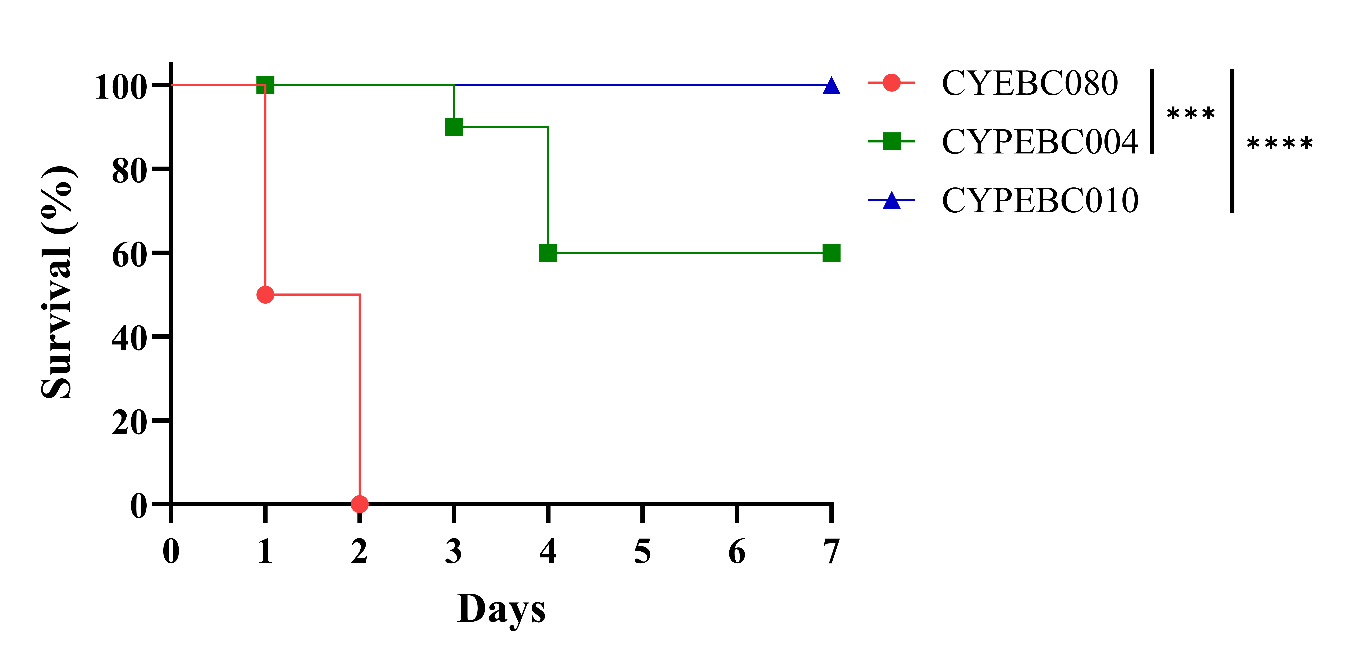


**Fig. S5. Analysis of the impact of phage treatment on the survival rate of larvae infected with CYEBC080.** **(A)** Morphology group 1 phages, **(B)** Morphology group 2 phages, and **(C)** Morphology group 3 phages. Larvae survival rates were monitored for up to seven days post-infection, with 10 larvae per group. The red circles represent the control group, where larvae were infected with CYEBC080 without phage treatment. The Mantel-Cox test was used to assess differences in overall larvae survival. All statistical analyses were performed relative to the untreated bacteria-only group (****, *p* < 0.0001; ***, *p* < 0.001; **, *p* < 0.01; ns, not significant).

**Figure S6.**

**(A).**


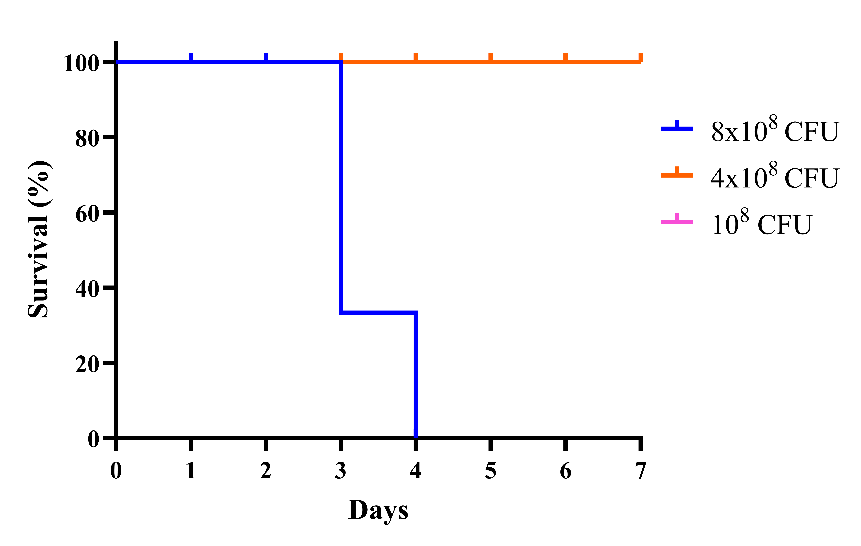


**(B).**


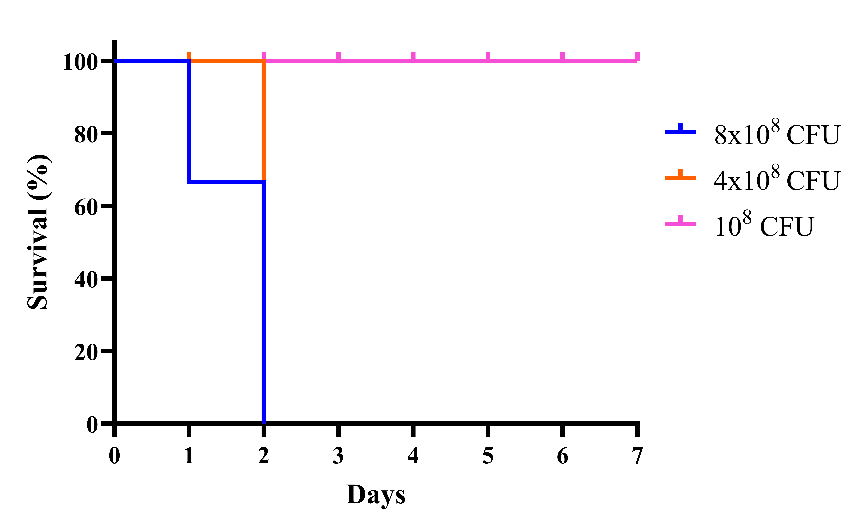


**Fig. S6. Determination of the optimal CFU for CYEBC080 (A) and CYEBC023 (B) used in the murine bacteremia model.** C57BL/6 mice were intraperitoneally inoculated with 10⁸, 4 × 10⁸, and 8 × 10⁸ CFU per mouse (3 mice per group), and survival was monitored for up to 7 days post-infection.

**Figure S7.**

**(A).**


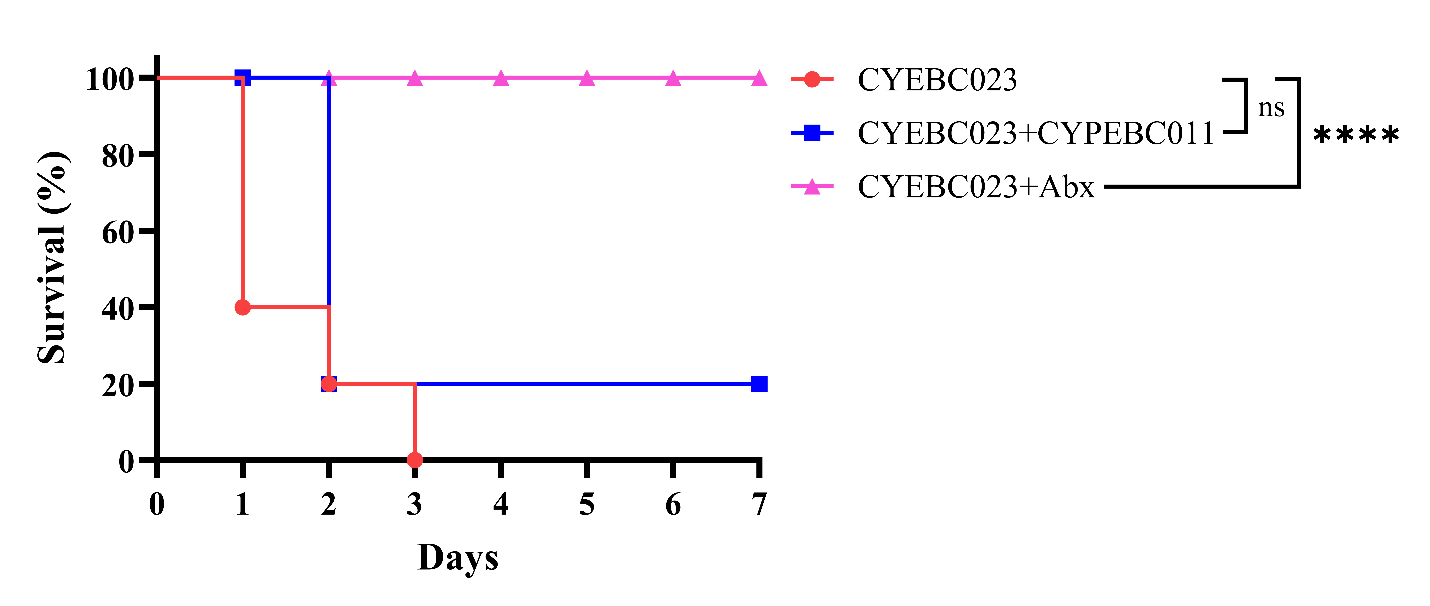


**(B).**


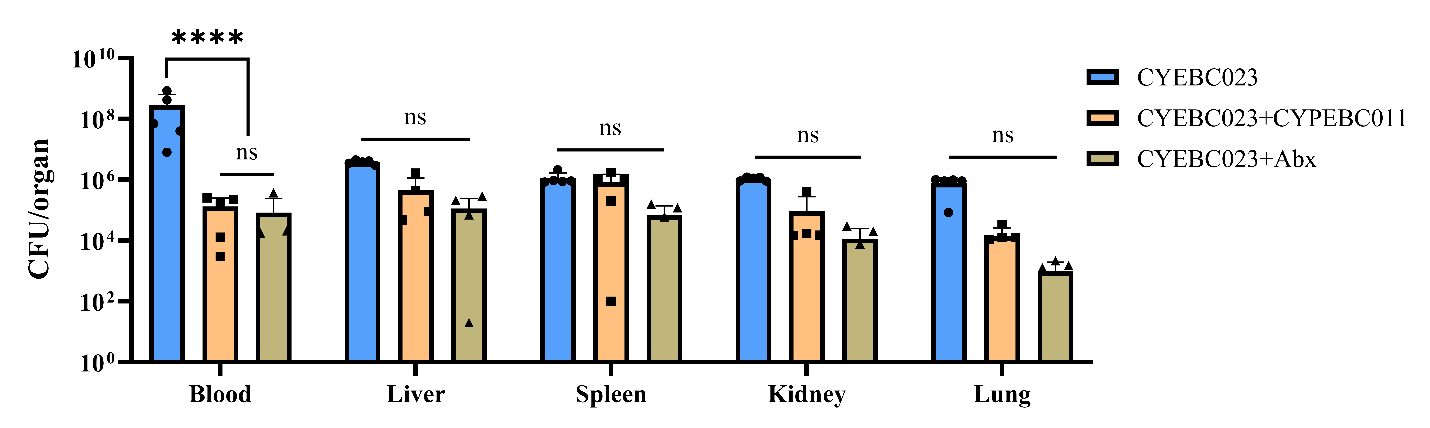


**(C).**


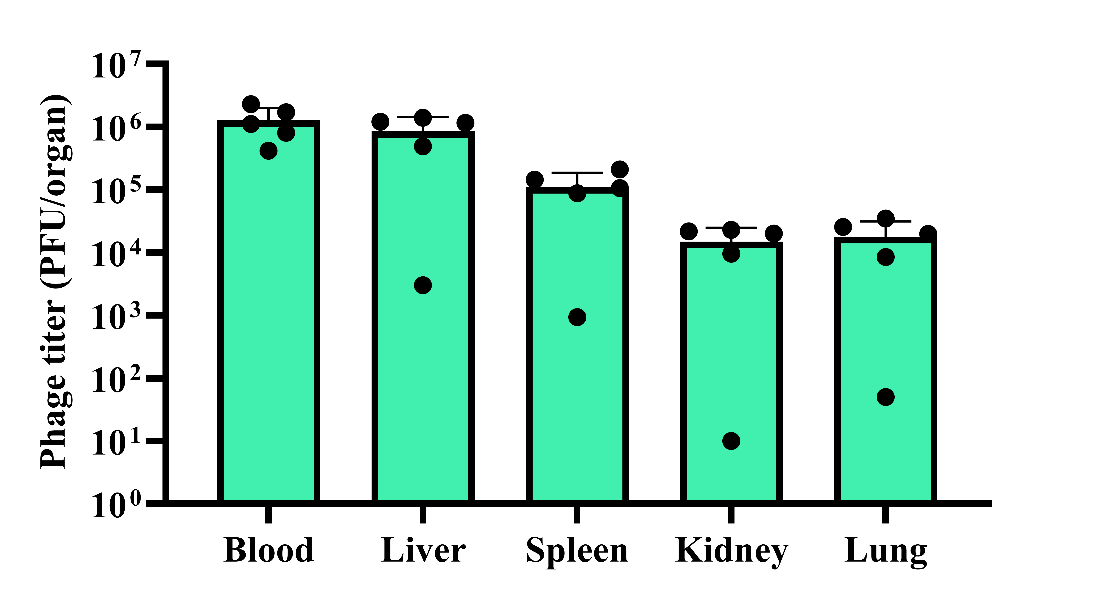


**Fig. S7. Therapeutic efficacy of phage CYPEBC011 in CYEBC023-infected mice. (A)**. Mouse survival, **(B)** bacterial load, and **(C)** phage titer following phage treatment in CYEBC023-infected mice. C57BL/6 mice were intraperitoneally inoculated with phage, bacteria, or both (n = 5 mice per group). **(A, B)** The CYEBC023-only group, which received bacterial infection without treatment, served as the control. The CYEBC023+CYPEBC011 group received phage treatment (MOI 10) one hour post-infection. The CYEBC023+Abx group received amikacin treatment (6 mg/kg) one hour post-infection. Mouse survival was monitored for up to 7 days. **(B, C)** Five mice from each group were sacrificed after 16 hours to assess bacterial burden and phage titers. Statistical analysis was performed using one-way ANOVA with Tukey’s multiple comparisons test. Significant differences (****, *p* < 0.0001; ns, not significant) are indicated by asterisks.

**Reference**

1. Ackermann HW. 2012. Bacteriophage electron microscopy. Adv Virus Res 82:1-32.

2. Ali SF, Teh SH, Yang HH, Tsai YC, Chao HJ, Peng SS, Chen SC, Lin LC, Lin NT. 2024. Therapeutic Potential of a Novel Lytic Phage, vB_EclM_ECLFM1, against Carbapenem-Resistant Enterobacter cloacae. Int J Mol Sci 25.

3. Zhu Y, Shang J, Peng C, Sun Y. 2022. Phage family classification under Caudoviricetes: A review of current tools using the latest ICTV classification framework. Front Microbiol 13:1032186.

4. Parmar K, Fackler JR, Rivas Z, Mandrekar J, Greenwood-Quaintance KE, Patel R. 2024. A comparison of phage susceptibility testing with two liquid high-throughput methods. Front Microbiol 15:1386245.

5. Rajnovic D, Munoz-Berbel X, Mas J. 2019. Fast phage detection and quantification: An optical density-based approach. PLoS One 14:e0216292.

6. Chen PK, Liu CY, Kuo HY, Lee YT, Liu YH, Zhang YZ, Kao CY. 2024. Emergence of extensively-drug-resistant hypervirulent Acinetobacter baumannii isolated from patients with bacteraemia: bacterial phenotype and virulence analysis. Int J Antimicrob Agents 64:107358.
