# Supplementary material for: Isolation and characterization of strictly lytic bacteriophages against carbapenem-resistant Enterobacter cloacae complex
Source: Microbiol Spectr. 2025 Oct 1;13(11):e00835-25. doi: 10.1128/spectrum.00835-25 (PMC12584729; doi:10.1128/spectrum.00835-25)
Supplement: Supplemental tables — Tables S1 to S5. [file spectrum.00835-25-s0002.docx]

**Table S1. Species and carbapenemase genes of *Enterobacter cloacae* complex isolates used in this study.**

| Bacterial strain | Species ID^a^ | NG-Test CARBA5 |
| --- | --- | --- |
| CYEBC001 | *E. cloacae* | IMP |
| CYEBC002 | *E. cloacae* | IMP, NDM |
| CYEBC003 | *E. cloacae* | IMP |
| CYEBC004 | *E. cloacae* | IMP |
| CYEBC005 | *E. asburiae* | IMP |
| CYEBC006 | *E. asburiae* | IMP |
| CYEBC007 | *E. asburiae* | IMP |
| CYEBC008 | *E. asburiae* | - |
| CYEBC009 | *E. cloacae* | IMP |
| CYEBC010 | *E. cloacae* | IMP |
| CYEBC011 | *E. cloacae* | - |
| CYEBC012 | *E. cloacae* | IMP |
| CYEBC013 | *E. cloacae* | IMP |
| CYEBC014 | *E. cloacae* | - |
| CYEBC015 | *E. cloacae* | - |
| CYEBC016 | *E. kobei* | - |
| CYEBC017 | *E. cloacae* | NDM |
| CYEBC018 | *E. cloacae* | - |
| CYEBC019 | *E. cloacae* | - |
| CYEBC020 | *E. asburiae* | - |
| CYEBC021 | *E. cloacae* | - |
| CYEBC022 | *E. cloacae* | - |
| CYEBC023 | *E. cloacae* | - |
| CYEBC024 | *E. cloacae* | - |
| CYEBC025 | *E. kobei* | - |
| CYEBC026 | *E. cloacae* | IMP |
| CYEBC027 | *E. asburiae* | - |
| CYEBC028 | *E. asburiae* | - |
| CYEBC029 | *E. cloacae* | - |
| CYEBC030 | *E. cloacae* | - |
| CYEBC031 | *E. cloacae* | - |
| CYEBC032 | *E. cloacae* | IMP |
| CYEBC033 | *E. kobei* | - |
| CYEBC034 | *E. cloacae* | - |
| CYEBC035 | *E. cloacae* | - |
| CYEBC036 | *E. cloacae* | - |
| CYEBC037 | *E. asburiae* | - |
| CYEBC038 | *E. asburiae* | - |
| CYEBC039 | *E. cloacae* | - |
| CYEBC040 | *E. cloacae* | - |
| CYEBC041 | *E. cloacae* | - |
| CYEBC042 | *E. cloacae* | - |
| CYEBC043 | *E. cloacae* | - |
| CYEBC044 | *E. cloacae* | - |
| CYEBC045 | *E. cloacae* | - |
| CYEBC046 | *E. cloacae* | NDM |
| CYEBC047 | *E. cloacae* | - |
| CYEBC048 | *E. kobei* | IMP |
| CYEBC049 | *E. cloacae* | - |
| CYEBC050 | *E. cloacae* | - |
| CYEBC051 | *E. hormaechei* | - |
| CYEBC052 | *E. asburiae* | - |
| CYEBC053 | *E.hormaechei* | - |
| CYEBC054 | *E. cloacae* | NDM |
| CYEBC055 | *E. cloacae* | NDM |
| CYEBC056 | *E. cloacae* | NDM |
| CYEBC057 | *E. cloacae* | - |
| CYEBC058 | *E. asburiae* | - |
| CYEBC059 | *E. cloacae* | IMP |
| CYEBC060 | *E. cloacae* | - |
| CYEBC061 | *E. cloacae* | - |
| CYEBC062 | *E. asburiae* | - |
| CYEBC063 | *E. cloacae* | IMP |
| CYEBC064 | *E. cloacae* | NDM |
| CYEBC065 | *E. cloacae* | NDM |
| CYEBC066 | *E. cloacae* | - |
| CYEBC067 | *E. asburiae* | - |
| CYEBC068 | *E. kobei* | - |
| CYEBC069 | *E. cloacae* | - |
| CYEBC070 | *E. hormaechei* | - |
| CYEBC071 | *E. cloacae* | NDM |
| CYEBC072 | *E. cloacae* | - |
| CYEBC073 | *E. cloacae* | - |
| CYEBC074 | *E. cloacae* | - |
| CYEBC075 | *E. cloacae* | - |
| CYEBC076 | *E. cloacae* | IMP |
| CYEBC077 | *E. asburiae* | - |
| CYEBC078 | *E. cloacae* | - |
| CYEBC079 | *E. kobei* | - |
| CYEBC080 | *E. cloacae* | NDM |

IMP (Imipenemase), NDM (New Delhi Metallo-β-lactamase)

^a^ Species was identified by using MALDI-TOF MS. However, CYEBC023 and CYEBC080 were further determined as *Enterobacter hormaechei* by whole-genome sequencing.

**Table S2. Genomic characteristics of** ***Enterobacter hormaechei*** **CYEBC023 and CYEBC080.**

| Isolate | Chromosome/Plasmid | NCBI nucleotide assession number | Length (bp) | Replicon^a^ | Virulence gene^b^ | Antibiotic resistance gene^c^ |
| --- | --- | --- | --- | --- | --- | --- |
| CYEBC023 | Chromosome | CP180424 | 5117169 | - | *iroN*, *nlpI* | *bla*_ACT-15_, *fosA* |
|  | plasmid | CP180425 | 288681 | IncHI2-IncHI2A | *terC* | *aph(6)-Id*, *aph(3'')-Ib*, *aadA2b*, *aac(6')-IIc*, *bla*_SHV-12_, *bla*_TEM-1B_, *ere(A)*, *qnrB2*, *sul1*, *sul2*, *dfrA19* |
|  | plasmid | CP180428 | 6372 | - | - | - |
| CYEBC080 | Chromosome | CP181076 | 4742267 | - | *astA*, *nlpI* | *bla*_ACT-14_, *bla*_CTX-M-15_, *fosA* |
|  | plasmid | CP181077 | 309605 | IncHI2-IncHI2A | *terC* | *aac(3)-IIa*, *aph(6)-Id*, *aph(3'')-Ib*, *aadA1*, *aac(6')-Ib-cr*, *bla*_CTX-M-15_, *bla*_TEM-1B_, *bla*_OXA-1_, *bla*_NDM-1_, *bla*_OXA-10_, *catA1*, *catB3*, *qnrB1*, *ARR-3*, *sul1*, *sul2*, *tet(A)*, *dfrA27* |
|  | plasmid | CP181078 | 59234 | IncR | - | - |
|  | plasmid | CP181079 | 4961 | Col(pHAD28) | - | - |

^a^Plasmid replicon was determined by PlasmidFinder.

^b^Virulence genes were detected by virulence factor database (VFDB).

^c^Antibiotic resistance genes were found by using ResFinder.

**Table S3. Antibiotic resistance profiles of carbapenem-resistant *E. cloacae* complex CYEBC023 and CYEBC080.**

|  | Ampicillin | Amoxicillin-clavulanate | Ampicilin-sulbactam | Piperacillin-ticarcillin | Cefazolin | Cefepime | Ceftriaxone | Cefuroxime | Ceftazidime | Cefixime | Aztreonam | Doripenem | Ertapenem |
| --- | --- | --- | --- | --- | --- | --- | --- | --- | --- | --- | --- | --- | --- |
| CYEBC023 | R | R | R | R | R | S | R | R | R | R | R | R | R |
| CYEBC080 | R | R | R | R | R | R | R | R | R | R | R | I | R |

|  | Meropenem | Imipenem | Colistin | Polymyxin B | Gentamicin | | Tobramicin | Amikacin | Tetracycline | Ciprofloxacin | Levofloxacin | Trimethorpim-sulfamethoxazole | Chloramphenicol |
| --- | --- | --- | --- | --- | --- | --- | --- | --- | --- | --- | --- | --- | --- |
| CYEBC023 | R | S | S | S | | R | R | I | S | I | I | R | R |
| CYEBC080 | R | R | S | S | | R | R | S | R | R | R | R | I |

Antibiotic resistance was determined by the disk diffusion test method. ‘Susceptible’, ‘Intermediate’, and ‘Resistant’ are abbreviated as ‘S’, ‘I’, and ‘R’.

**Table S4. Characteristics of isolated phages in this study.**

| Phage ID | RAPD pattern (regroup)^a^ | HL/HW/TL/TW tail (nm)^b^ | Plaque size (mm) | Host bacteria (CFU/mL) | Phage titer (PFU/mL) | MOI | Source of isolation | Collection date (mm/dd/yyyy) | NCBI biosample  accession number |
| --- | --- | --- | --- | --- | --- | --- | --- | --- | --- |
| CYPEBC001 | G1 (2) | 114/81/111/21 | 0.2-1 | 10^6^ | 10^8^ | 6.67+2.89 | Factory wastewater-Beitou | 12/16/2022 | SAMN46521311 |
| CYPEBC002 | G2 (-) | - | 0.2-1 | 10^6^ | 3 x 10^8^ | ND | Factory wastewater-Beitou | 12/16/2022 | - |
| CYPEBC003 | G3 (2) | 110/78/103/21 | 0.2-1 | 10^6^ | 4 x 10^8^ | 4.17+ 1.44 | Stream water-Beitou | 12/16/2022 | SAMN46528651 |
| CYPEBC004 | G4 (3) | 109/83/104/20 | 0.2-1 | 10^6^ | 5 x 10^8^ | 0.07+ 0.02 | Hospital wastewater-Kaohsiung | 2/6/2023 | SAMN46529921 |
| CYPEBC005 | G5 (-) | - | 0.2-1 | 10^6^ | 5 x 10^8^ | ND | Hospital wastewater-Kaohsiung | 2/6/2023 | - |
| CYPEBC006 | G6 (2) | 109/84/97/25 | 0.2-1 | 10^6^ | 10^8^ | 0.07+ 0.02 | Hospital wastewater-Kaohsiung | 5/26/2023 | SAMN46529922 |
| CYPEBC007 | G7 (1) | 98/79/114/24 | 0.2-1 | 10^6^ | 10^8^ | 6.67+2.89 | Hospital wastewater-Kaohsiung | 5/26/2023 | SAMN46529967 |
| CYPEBC008 | G5 (1) | 99/73/117/22 | 0.2-1 | 10^6^ | 10^9^ | 2.92+1.91 | Hospital wastewater-Kaohsiung | 5/26/2023 | SAMN46529969 |
| CYPEBC009 | G8 (-) | - | 0.2-1 | 10^6^ | 6 x 10^9^ | ND | Domestic wastewater-Beitou | 6/30/2023 | - |
| CYPEBC010 | G9 (3) | 102/77/108/22 | 0.2-1 | 10^6^ | 2 x 10^8^ | 0.26+0.09 | Domestic wastewater-Beitou | 6/30/2023 | SAMN46529979 |
| CYPEBC011 | G2 (2) | 109/88/106/23 | 0.2-1 | 10^6^ | 8 x 10^8^ | 0.26+0.09 | Stream water-Beitou | 7/2/2023 | SAMN46529980 |
| CYPEBC012 | G8 (1) | 93/69/109/22 | 0.2-1 | 10^6^ | 8 x 10^9^ | 0.13+0.05 | Stream water-Beitou | 7/2/2023 | SAMN46529989 |
| CYPEBC013 | G10 (-) | - | 0.2-1 | 10^6^ | 2 x 10^9^ | ND | Stream water-Beitou | 7/2/2023 | - |
| CYPEBC014 | G10 (2) | 115/80/103/29 | 0.2-1 | 10^6^ | 8 x 10^8^ | 0.13+0.16 | Stream water-Beitou | 7/2/2023 | SAMN46530063 |
| CYPEBC015 | G11 (2) | 104/76/109/20 | 0.2-1 | 10^6^ | 10^9^ | 2.92+1.91 | Stream water-Beitou | 9/15/2023 | SAMN46530450 |
| CYPEBC018 | G12 (1) | 84/54/117/15 | 0.2-1 | 10^6^ | 5 x 10^9^ | 0.09+0.06 | Stream water-Beitou | 9/15/2023 | SAMN46530451 |
| CYPEBC020 | G10 (-) | - | 0.2-1 | 10^6^ | 10^9^ | ND | Stream water-Beitou | 9/20/2023 | - |
| CYPEBC021 | G10 (-) | - | 0.2-1 | 10^6^ | 10^9^ | ND | Stream water-Beitou | 9/20/2023 | - |

^a^The phages were reclassified into three groups according to their head and tail sizes.

^b^Morphological properties of isolated phages were measured using imageJ.

HL, head length; HW, head width; TL, tail length; TW, tail width; RAPD, Random amplified polymorphic DNA; MOI: Multiplicity of infection; ND, not determined.

**Table S5. Average Nucleotide Identity comparison between the 12 phage genomes and four well-characterized *Enterobacter* phages, ENC22, Entb_43, fGh-Ecl01, and fGh-Ecl01.**

|  | **Average Nucleotide Identity (%)** | | | |
| --- | --- | --- | --- | --- |
| **Phage ID** | **ENC22** | **Entb_43** | **fGh-Ecl01** | **fGh-Ecl04** |
| **CYPEBC001** | 91.8763 | 80.1407 | 80.0595 | 80.259 |
| **CYPEBC003** | 91.7937 | 80.315 | <70 | <70 |
| **CYPEBC004** | 91.7898 | 80.0458 | 79.8674 | 80.1378 |
| **CYPEBC006** | 91.7327 | <70 | <70 | 80.03 |
| **CYPEBC007** | 91.7359 | 80.4019 | 80.0783 | 80.232 |
| **CYPEBC008** | 91.8123 | <70 | 79.8859 | 80.1081 |
| **CYPEBC010** | 91.7167 | 79.9928 | 79.7639 | 79.8648 |
| **CYPEBC011** | 91.8937 | 79.7894 | 79.8207 | 79.8546 |
| **CYPEBC012** | 91.7615 | 80.3906 | 80.227 | 80.2472 |
| **CYPEBC014** | 91.5779 | <70 | <70 | <70 |
| **CYPEBC015** | 91.5569 | <70 | <70 | 80.072 |
